# Genetic and Structure-Function Studies of Missense Mutations in Human Endothelial Lipase

**DOI:** 10.1371/journal.pone.0055716

**Published:** 2013-03-25

**Authors:** Hamid Razzaghi, Anna Tempczyk-Russell, Kurt Haubold, Stephanie A. Santorico, Touraj Shokati, Uwe Christians, Mair E. A. Churchill

**Affiliations:** 1 Division of Cardiology, Department of Medicine, University of Colorado Denver, Aurora, Colorado, United States of America; 2 Accelrys, Inc., San Diego, California, United States of America; 3 Department of Mathematical and Statistical Sciences, University of Colorado Denver, Denver, Colorado, United States of America; 4 Department of Anesthesiology, University of Colorado Denver, Aurora, Colorado, United States of America; 5 Department of Pharmacology, University of Colorado Denver, Aurora, Colorado, United States of America; University of Bonn, Institut of Experimental Hematology and Transfusion Medicine, Germany

## Abstract

Endothelial lipase (EL) plays a pivotal role in HDL metabolism. We sought to characterize EL and its interaction with HDL as well as its natural variants genetically, functionally and structurally. We screened our biethnic population sample (n = 802) for selected missense mutations (n = 5) and identified T111I as the only common variant. Multiple linear regression analyses in Hispanic subjects revealed an unexpected association between T111I and elevated LDL-C (p-value = 0.012) and total cholesterol (p-value = 0.004). We examined lipase activity of selected missense mutants (n = 10) and found different impacts on EL function, ranging from normal to complete loss of activity. EL-HDL lipidomic analyses indicated that EL has a defined remodeling of HDL without exhaustion of the substrate and a distinct and preference for several fatty acids that are lipid mediators and known for their potent pro- and anti-inflammatory properties. Structural studies using homology modeling revealed a novel α/β motif in the C-domain, unique to EL. The EL dimer was found to have the flexibility to expand and to bind various sizes of HDL particles. The likely impact of the all known missense mutations (n = 18) on the structure of EL was examined using molecular modeling and the impact they may have on EL lipase activity using a novel structure-function slope based on their structural free energy differences. The results of this multidisciplinary approach delineated the impact of EL and its variants on HDL. Moreover, the results suggested EL to have the capacity to modulate vascular health through its role in fatty acid-based signaling pathways.

## Introduction

A long list of epidemiological studies and prospective randomized trials has consistently shown a strong inverse relationship between the levels of high-density lipoprotein cholesterol (HDL-C) and the risk of coronary heart disease (CHD). Population studies also demonstrate that for each 1 mg/dl increase in HDL-C level, cardiovascular mortality is decreased by 2–3% [1]. However, in addition to absolute plasma levels, recent data suggest that the biological functions of HDL are important in assessing the role of HDL in CHD and major cardiovascular events [2], [3]. HDL particles have atheroprotective properties, including reverse cholesterol transport, anti-inflammatory, anti-oxidant, anti-thrombotic, and nitric oxide promoting effects [4]. Low HDL-C (hypoalphalipoproteinemia), which is re-defined as below 40 mg/dl for both men and women, is therefore, a major CHD risk factor [5]. The prevalence of low HDL-C in the U.S. adult population was 41.8 million (8.9%) in 2008 [6]. Low HDL-C was even predictive of risk of major cardiovascular events in statin-treated patients who had low-density lipoprotein cholesterol (LDL-C) below 70 mg/dl [7]. Therefore, understanding factors contributing to HDL homeostasis is of great interest for public health. One of the key factors affecting HDL metabolism is the enzyme, endothelial lipase, for which HDL is a preferred substrate.

Endothelial lipase (*LIPG* gene, EL protein) is a unique member of the plasma triglyceride lipase family, which includes lipoprotein lipase (LPL) and hepatic lipase (HL). Features that make EL unique include its expression by vascular endothelial cells and up-regulation of its expression by inflammatory cytokines such as TNFα and IL-1β [8], [9], [10]. Since neither LPL nor HL is expressed by endothelial cells, and both are down-regulated by the inflammatory cytokines, EL may have a special role in vascular endothelium when it is under an inflammatory condition, such as atherosclerosis. Indeed, further functional studies confirmed a relationship of EL with inflammation [11], [12].

**Figure 1:**
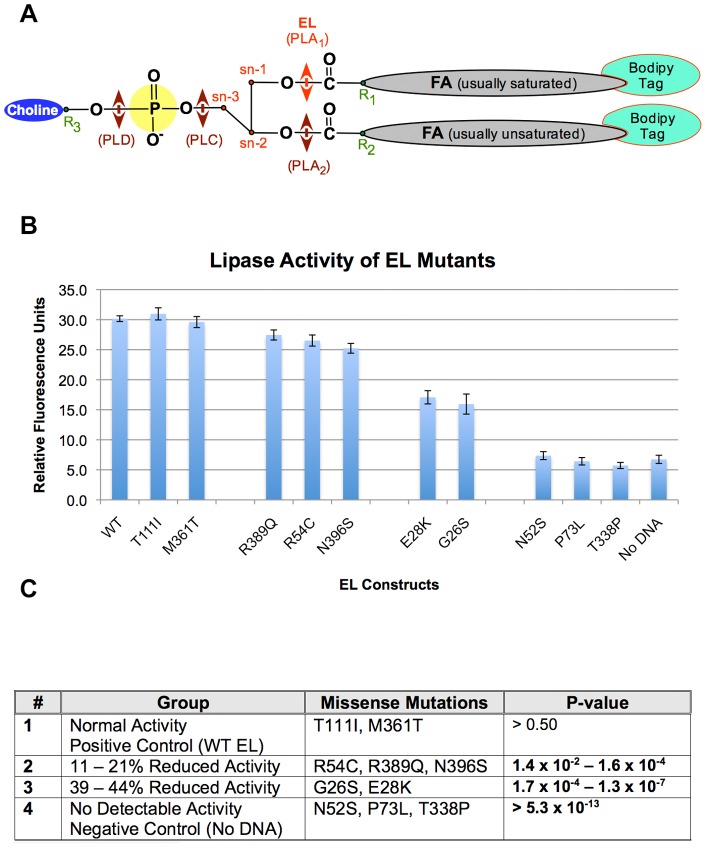
Schematic diagram of a fluorescent-labeled phospholipid (phosphatidylcholine) used in the lipase assay and the site of cleavage by EL (A). Determination of phospholipase activity of wild type and ten mutant EL variants (**B**). Standard error of means (SEM) is provided for the graph. The p-values are obtained from comparison between each group with the positive control using ANOVA (**C**).

Through analysis of EL interaction with reconstituted HDL_3_ (rHDL_3_), Gauster et al. [13] showed EL has serine-phospholipase A1 (PLA_1_) and lysophospholipase activities but no PLA_2_ activity (**Figure 1A**). EL releases both sn-1 and sn-2 fatty acids (FAs) from phospholipids of HDL particles. However, because EL does not have sn-2 phospholipase activity, sn-2 FA must first transfer to the sn-1 position, which occurs spontaneously, before EL can liberate the relocated sn-2 FA through its lysophospholipase activity [13]. Furthermore, EL can liberate both saturated and unsaturated fatty acids from HDL, but displays variable cleavage efficiency, depending on the length of fatty acid and the degree of saturation. Gauster et al. [13] suggested EL hydrolyzes shorter and more saturated FAs more efficiently than those with longer acyl chains and less saturated FAs. In addition to its phospholipase activity, EL has very low but detectable triacylglycerol lipase activity [14].

Independent of its lipase activity, EL, like LPL and HL, also has a “bridging function” (ligand-binding function), which involves binding plasma lipoproteins via the heparan sulfate proteoglycan (HSPG)-mediated pathway, leading to lipoprotein cellular uptake and degradation [15]. However, EL has a distinctive bridging preference that is different from LPL and HL. EL is far more efficient in bridging VLDL and LDL than HL or LPL. Furthermore, bridging LDL results in its uptake and degradation, while bridging HDL results in uptake with the majority being returned to plasma [15]. Since LPL does not bridge HDL, this nonlipolytic function may be another way EL and HL influence HDL homeostasis. However, the bridging function of EL may have an adverse effect on vascular wall health. Under inflammatory conditions, EL may act as a bridging molecule between circulating monocytes and the vessel wall, leading to monocyte recruitment into sub-endothelial spaces, an early event in atherogenesis [11].

Tertiary structure of EL is not known yet. However, based on homology modeling with X-ray crystallographic-determined 3D structure of pancreatic lipase, it is possible to deduce common structural features for members of the plasma lipase family. For EL, these include a putative two-domain conformation, triad catalytic site (S^169^, D^193^, H^274^) covered by a lid structure, a lipid-binding motif, and a heparin-binding motif [**Files S2 and S4 in Supporting Information S1**].

Changes in the levels of EL expression have a profound impact on HDL metabolism. Animal studies with transgenic mice over-expressing EL and with EL knockout mice showed a significant decrease and elevated increase in HDL-C levels, respectively [8], [10], [16], [17], [18]. In human studies, several missense mutations have been identified that affect EL activity and HDL-C levels [19], [20].

Because of its role in HDL metabolism, *LIPG* gene has been subjected to intensive screening for mutations and genetic associations in several population, epidemiological, and clinical studies [18], [21], [22], [23], [24], [25], [26], [27], [28]. Altogether, there are currently 18 missense mutations listed in the published papers or deposited in the NCBI SNP database: G26S, E28K, N52S, R54C, P73L, T111I, A116T, G176R, I239T, T298S, C311Y, T338P, M342V, M361T, R389Q, N396S, R476Q, and R476W. The resulting structural variations in EL may have different impacts on HDL composition and function, which in turn, may alter the risk for atherosclerosis.

We have investigated the genetics, structure, function, and lipidomics of the EL-HDL interaction; our results provide a platform for comprehensive and meaningful interpretation of the impact coding variations in this gene may have on raising HDL levels therapeutically or on the risk of atherosclerosis in the general population.

## Results

We subjected selected missense mutations in the human *LIPG* to genetic, functional, lipidomics and structural studies. The details of these studies are provided in the following sections.

### Genetic Association of the Mutations

#### LIPG-T111I variant is associated with total cholesterol and LDL-C levels in Hispanics

The occurrence of *LIPG* missense mutations, G26S, E28K, N52S, R54C, and T111I, which were selected as representatives of each of the four lipase activity level groups (**Figure 1**), was determined in the available SLVDS samples (n = 802, Whites = 426, Hispanics = 376) using pyrosequencing. We did not find any subjects with G26S, E28K, or R54C mutations in our samples. Only one subject was identified with N52S mutation [minor allele frequency (MAF) = 0.0008], who was a 58-year old white female with total HDL-C of 74 mg/dl. Therefore, no association analysis was possible. We found T111I to be a common polymorphism in our population sample, with slightly higher frequency in Whites (MAF = 0.276) than in Hispanics (MAF = 0.223) (**Table 1**). The frequency of T111I varies from one population to another. Its frequency is the lowest in Sub-Saharan Africa (MAF = 0.034) and highest in Asia (MAF = 0.409) (ref SNP ID: rs2000813, NCBI SNP database). The details of the lipid panel (cholesterol, triglyceride, total HDL, HDL_2_, HDL_3_, LDL) in our subjects with T111I missense are provided (**File S1 in Supporting Information S1**). Multiple regression analyses did not identify an association between HDL-C levels and the minor allele (T). Instead, we found a significant association with total cholesterol and LDL-C levels in Hispanics (**Table 2**), with a CC ⇒ TC ⇒ TT genotypic increase in the levels of cholesterol and LDL-C. This is evident from the regression coefficient (B-value, which indicates the amount by which the tested variable is changed) being higher for TT than for TC. For example, mean LDL-C levels in Hispanics for the T111I genotypes were: CC = 130.48 mg/dl, TC = 136.28 mg/dl, TT = 156.39 mg/dl; the overall average was 133.68 mg/dl. The B-value for the TT genotype was 27.4; lower bound of 95% confidence for B, 6.157, and upper bound, 48.670.

**Table 1:** Allelic and genotypic distribution of the *LIPG* T111I SNP in the bi-ethnic population samples of San Luis Valley in Colorado.

ETHNICITY	T111I Allelic Distribution			T111I Genotypic Distribution			
	C	T	Total	C/C	T/C	T/T	Total
WHITES	0.724	0.276	1.000	223(52.35%)	171(40.14%)	32(7.51%)	426(100%)
HISPANICS	0.777	0.223	1.000	224(59.57%)	136(36.17%)	16(4.26%)	376(100%)
Total				447	307	48	802

The genotypic frequencies are indicated in the parentheses.

**Table 2:** Multiple linear regression analyses of *LIPG* T111I SNP in the bi-ethnic population samples of San Luis Valley in Colorado.

Tested Variables	AllelicAssociation		GenotypicAssociation	
	WhitesP-value	HispanicsP-value	WhitesP-value	HispanicsP-value
**Total HDL-C**	T: 0.474	T: 0.117	TT: 0.266TC: 0.963	TT: 0.090TC: 0.411
**HDL_2_-C**	T: 0.401	T: 0.388	TT: 0.412TC: 0.629	TT: 0.112TC: 0.949
**HDL_3_-C**	T: 0.362	T: 0.134	TT: 0.199TC: 0.931	TT: 0.129TC: 0.391
**LDL-C**	T: 0.096	**T: 0.020**(B-value: 8.700)	TT: 0.087TC: 0.383	TT: 0.012*TC: 0.228
**Total Cholesterol**	T: 0.427	**T: 0.006**(B-value: 0.050)	TT: 0.390TC: 0.720	TT: 0.004*TC: 0.134
**Triglyceride**	T: 0.445	T: 0.826	TT: 0.775TC: 0.110	TT: 0.861TC: 0.870

*	Significant Dependent Variables (Hispanics)	Effect	B-value	Standard Error		P-value	95% CI(Lower, Upper)
	**LDL-C**	Intercept	93.792	42.150	**0.027**		(10.907, 176.677)
		Age	0.524	0.171	**0.002**		(0.187, 0.861)
		**TT**	27.414	10.810	**0.012**		(6.157, 48.670)
		**TC**	5.477	4.537	0.228		(−3.445, 14.398)
	**Total Cholesterol**	Intercept	5.155	0.044	**<0.001**		(5.068, 5.242)
		Age	0.003	0.001	**<0.001**		(0.002, 0.005)
		**TT**	0.152	0.052	**0.004**		(0.050, 0.255)
		**TC**	**0.033**	**0.022**	**0.134**		**(−0.010, 0.076)**

Multiple linear regression analyses of *LIPG* T111I SNP. The analyses were performed separately for Whites and Hispanics. The model is adjusted for gender, age, BMI, and smoking. The regression models were performed first for significant covariate(s) selection using stepwise variable selection. Then T111I SNP was added to the model that included significant covariates in an ‘enter’ method. Both genotypic and allelic association of the T111I with the lipid panel measurements were tested. For genotypic association, the genotypes were recoded in order to give weight to each genotype as following: CC = 0, CT = 1, TT = 2. For allelic association studies, dummy variables were created for the C/T alleles as following and both were included in the regression model: D_TT = 0 (for T/C and C/C genotypes); D_TT = 1 (for T/T genotype); D_TC = 0 (for T/T and C/C genotypes); D_TC = 1 (for T/C genotype). Similarly, dummy variables were created for covariate ‘smoking’ as following: D_Smoker = 0 (for nonsmokers and ex-smokers) and D_Smoker = 1 (for current smokers). B-value is the regression coefficient, which indicates the amount by which the tested variable is changed. The details of each significant linear regression model are provided under asterisk (*).

### Functional Analysis of the Mutations

#### Different missense mutations have different degrees of impact on EL's phospholipase activity

To date, 18 missense mutations have been reported for human *LIPG*. We tested ten of these for their efficiency in phospholipase activity, which was not normalized (**File S7 in Supporting Information S1**). The selection was based on earlier reports from the literature and online databases. We observed a wide range of efficiencies in their lipase activities, which could be clustered in four groups: The first group, T111I and M361T, had normal lipase activity similar to wild type EL. The second group, R54C, R389Q, and N396S, had 11–21% reduced activity. The third group, G26S and E28K, had 39–44% reduced activity. The fourth group, N52S, P73L, T338P, had no lipase activity above the level of negative control (**Figure 1B**). These results indicate that variations in EL may impact the rate at which HDL is remodeled in human.

### Lipidomics Analysis of the EL-HDL Interaction

#### EL hydrolyzes HDL-bound fatty acids in a defined and selective manner, which has implications for HDL homeostasis and inflammatory signaling pathways

**Figure 2:**
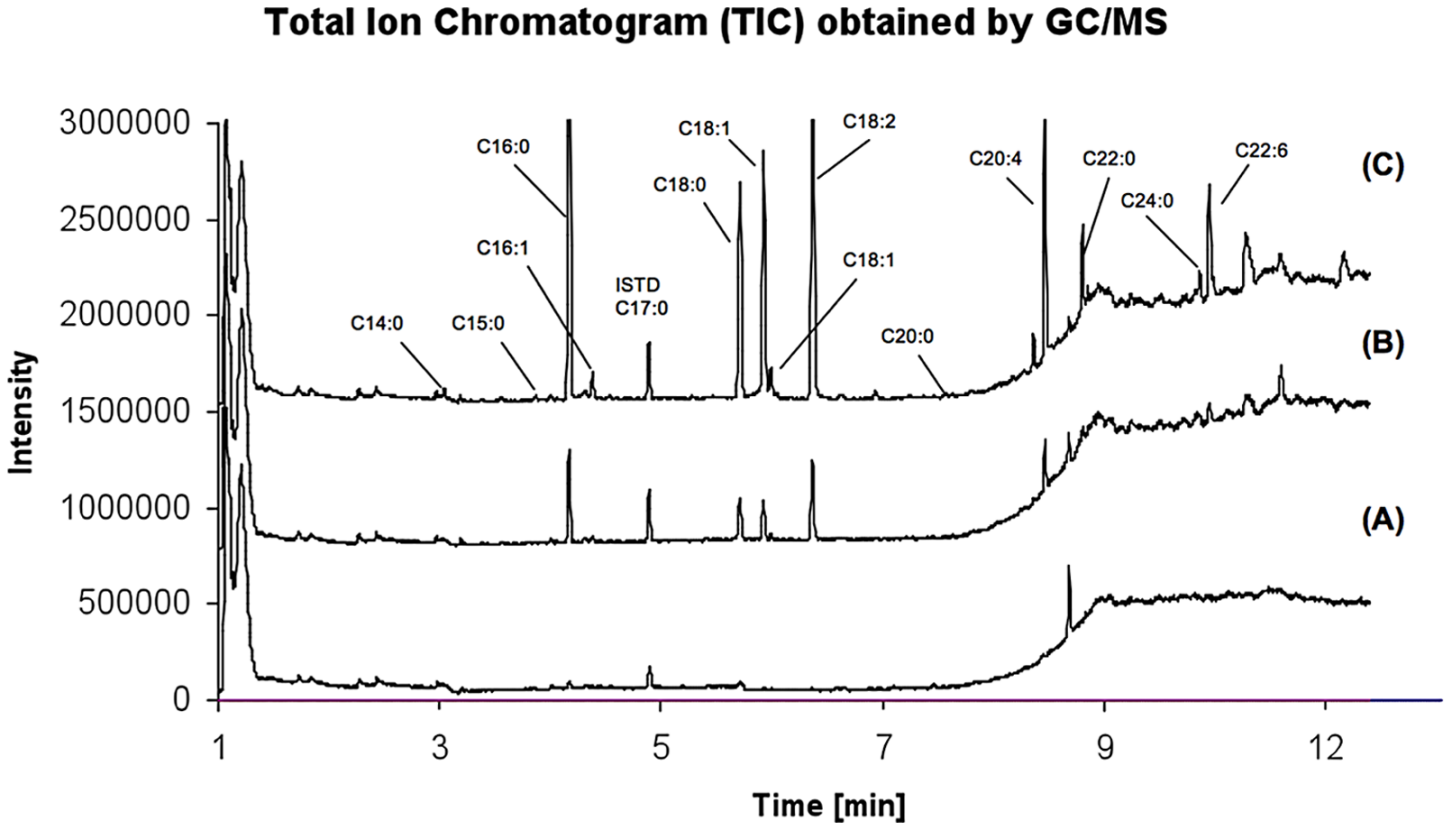
Total ion chromatograms (TIC) of HDL incubated with mutant (T338P) EL (A), HDL incubated with wild-type EL (B), and of untreated HDL (C). 40 µl of wild-type (C) and mutant samples (B) were individually added to 40 µl HDL samples (1 mg/ml). After an incubation time of 60 min at 37°C, 50 µl of 5 M NaCl was added to each sample in order to stop the reaction. The derivatization of samples was performed using methanolic acid and incubation at 120°C for 45 min. Fatty acids C18∶1 (cis-9) and C:18 (cis-11) are referred to the first and second C18∶1 peaks, respectively.

**Figure 3:**
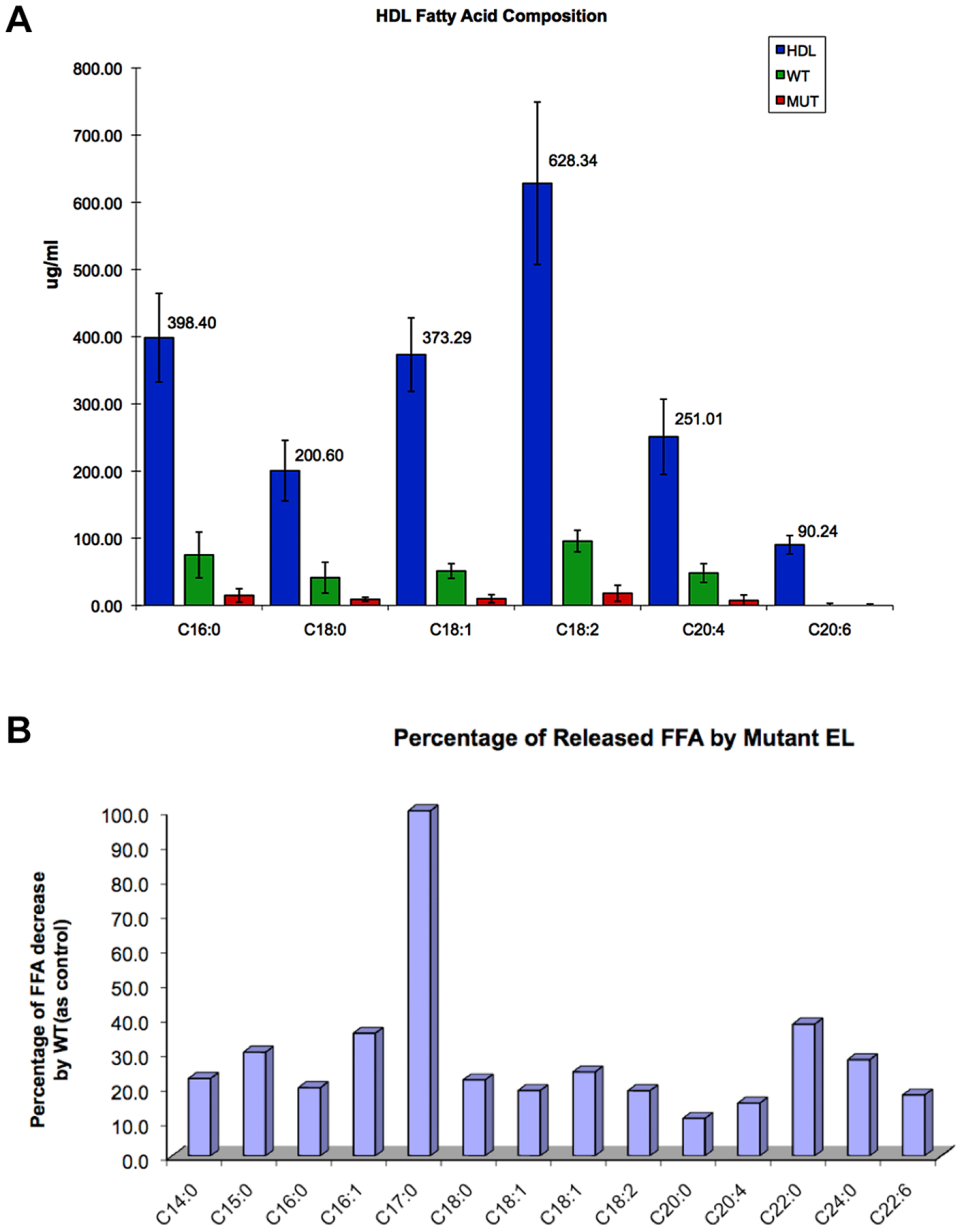
HDL fatty acid composition after hydrolysis of HDL by EL in mutant (T338P) and in wild-type (A). Released HDL fatty acid by mutant EL (labeled red, n =  3) was compared to released HDL fatty acid by wild type EL (labeled green, n =  3) and to free fatty acids composition in untreated HDL (labeled blue, n =  3). The percentage decrease of fatty acids released from HDL by mutant EL compared to wild type (**B**). The fatty acids liberated by EL were quantified by subtraction of untreated HDL (negative control) data.

**Figure 4:**
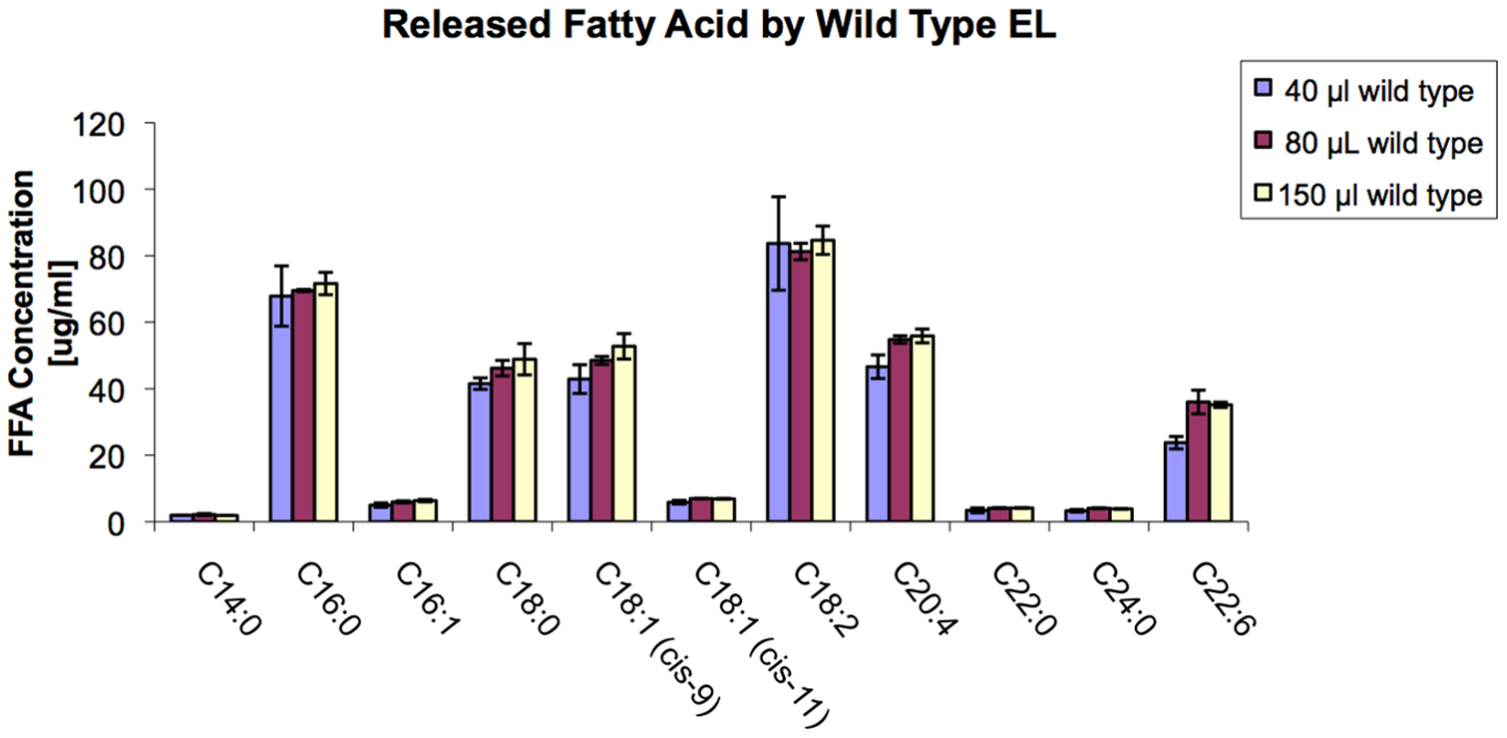
The effect of different EL-WT concentrations on HDL hydrolysis.

We sought to determine if there was a difference in the way HDL was hydrolyzed between wild type and mutant EL besides the obvious reaction rate. Gas chromatography/mass spectrometry (GC/MS) analyses showed that compared to wild type, EL-T338P mutant lost the ability to hydrolyze HDL-bound fatty acids substantially (**Figure 2**). This was consistent with the results obtained from our lipase assays. Comparisons between fatty acid composition of HDL and fatty acids released from HDL by wild type versus mutant EL revealed that the wild type and mutant released only a small, albeit differential, fraction of HDL fatty acids (**Figure 3A**). It was possible to compare the amount of the hydrolyzed specific fatty acids between wild type and mutant. For example, release of arachidonic acid (C20∶4) from HDL was reduced in the mutant by 80% whereas release of palmitoleic acid (C16∶1) by only 40% (**Figure 3B**); suggesting carriers of such mutations not only have impaired HDL remodeling but differential release of certain fatty acids. Overall, there was no significant difference in the amounts of fatty acids released from HDL by different EL concentration; indicating partial hydrolysis of HDL is independent of EL concentration (**Figure 4**). The exception was DHA-omega 3 (C22∶6) where its release was significantly higher at EL-WT 80 µl concentration than 40 µl. These results suggest that the lipase activity of EL does not lead to complete HDL catabolism but to HDL remodeling to smaller species. Furthermore, our total HDL lipidomic analyses indicated that EL has a distinct preference for certain fatty acids, with low preference for myristic acid (C14∶0), palmitoleic acid (C16∶1), cis-vaccenic acid (C18∶1), behenic acid (C22∶0), and lignoceric acid (C24∶0), and high preference for palmitic acid (C16∶0), stearic acid (C18∶0), oleic acid (C18∶1), linoleic acid (C18∶2), arachidonic acid (C20∶4), and docosahexaenoic acid (C22∶6, DHA-omega 3) (**Figures 2** and **4**). The selective release of fatty acids that are known lipid mediators, such as palmitic acid, linoleic acid, arachidonic acid, and docosahexaenoic acid, suggests that EL may modulate inflammatory signal pathways.

### Homology Modeling of EL

#### The C-domain in EL contains a novel structural motif that may be important for interaction with HDL

**Figure 5:**
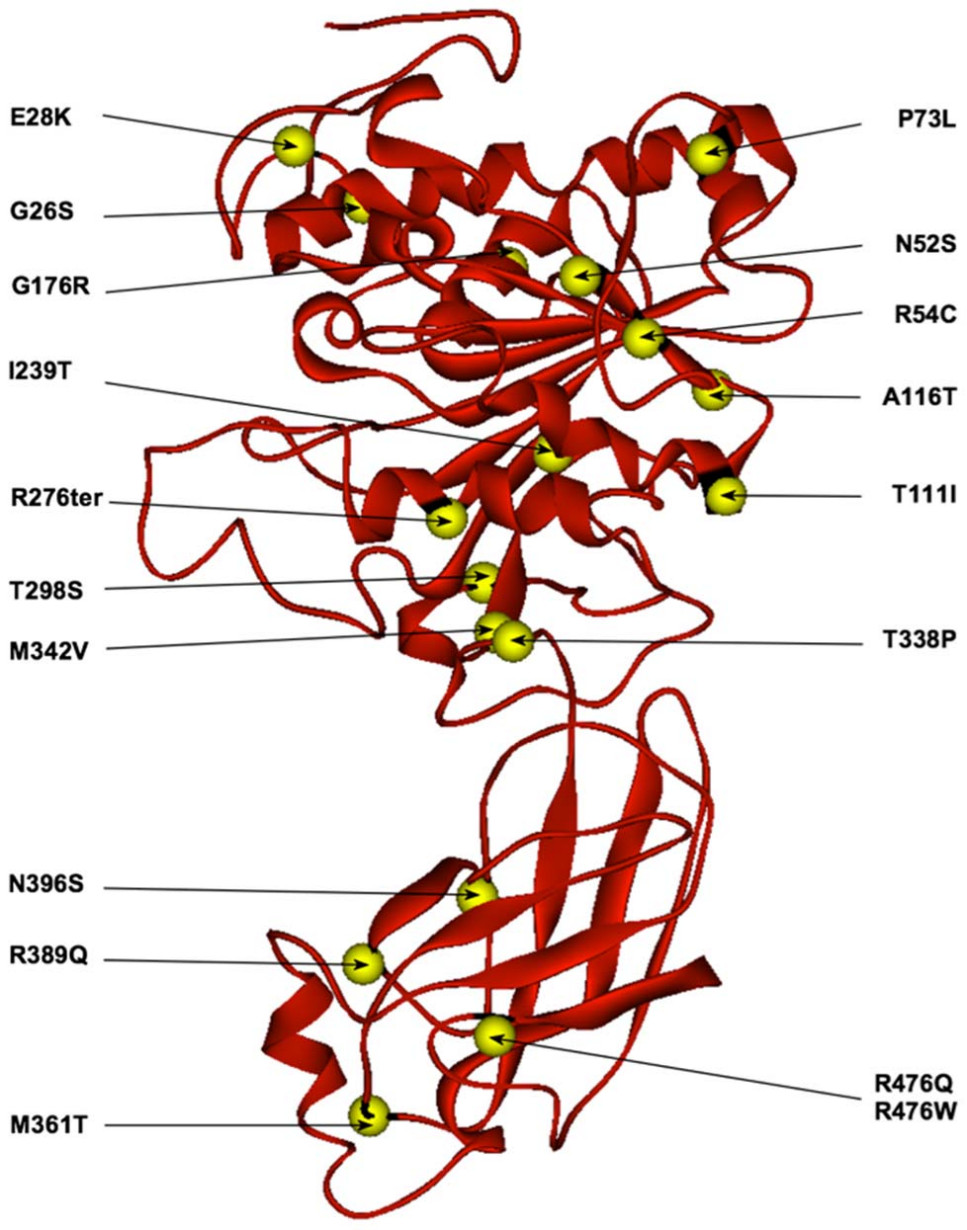
Molecular model of EL and the location of all the known missense mutations (to date) on the structure.

**Figure 6:**
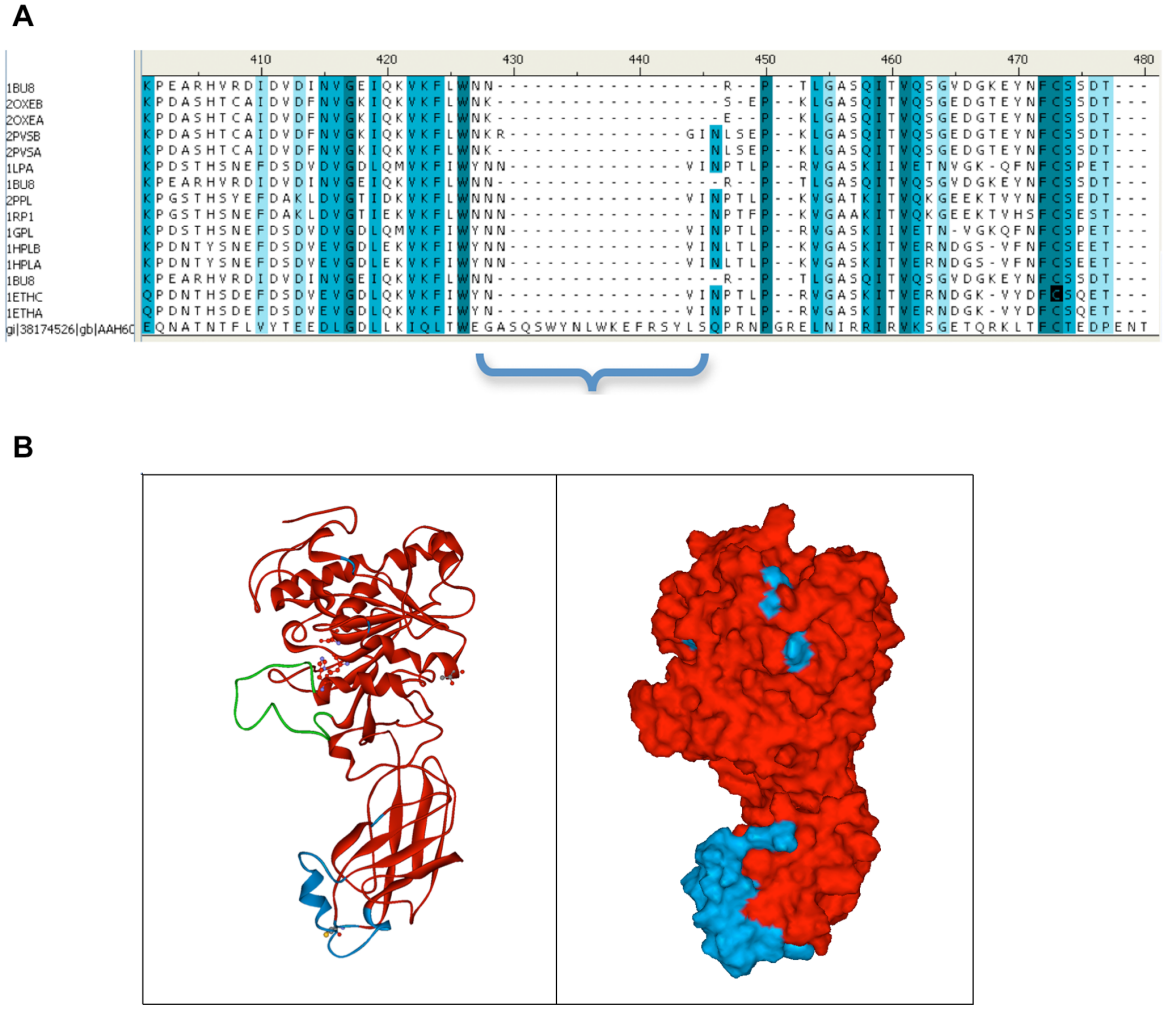
Identification of a unique structural motif in the C-domain of EL using multiple sequence alignment (A). The motif is 23-residue long and forms a coil/helix element, shown in blue (**B**).

We generated a molecular model of human EL based on its sequence homology to other lipases with known 3D structures. Our molecular modeling studies show that EL forms a two-domain protein with a lid structure covering the catalytic site in the N-domain (**Figure 5**). The N-terminal domain, which comprises residues 1–341, is an α/β type. It contains the catalytic triad (S^169^, D^193^, H^274^), which is responsible for hydrolysis of HDL phospholipids. Previous structural studies of lipases have delineated the molecular mechanism of catalytic triad function [29], [30], [31]. Briefly, the catalytic site is inaccessible to substrate in solution as long as the lid (252–272) covering the site is in closed conformation – the inactive state. In the active state, the protein assumes an open conformation, resulting in repositioning of the lid. The motion of the lid makes the active site accessible to the substrate, simultaneously forming a functional oxyanion hole and generating the lipase interfacial-binding site. In this study, we concentrated on the open, active conformation of the enzyme. The C-terminal domain of EL forms the PLAT domain – a stretch of 136 residues (347–482) – which is a β-sandwich composed of two-sheets of four strands each. The PLAT domain is involved in substrate binding and is found in a variety of lipid-associated proteins, including PL, LPL, HL, and EL [32]. Unlike other PLAT domains, the C-domain of EL contains an insertion of a novel motif of 23 residues (418– GASQSWYNLWKEFRSYLSQPRNPG –441). This motif is unique among the lipases, as no similar sequence or structure was found in any databases (**Figure 6A**). We predict this unique structural motif forms a coil/helix element in the C-domain of EL (**Figure 6B**). No mutation in this motif has ever been identified in population studies, suggesting that it may have an essential function in interacting with HDL particles. The two domains of EL are connected through a hinge loop (338–347), giving the molecule flexibility to bind to HDL in homodimer form. The hinge region contains a proteolytic cleavage site (RNKR) for proprotein convertase, a regulatory mechanism for EL function [33]. EL homodimer is formed by association of N-terminal amino acid (54– RTSKDPEHE –62) with the C-terminal amino acids from a second EL molecule D^378^, K^411^, and (476– QELWFRKC –483) in a head-to-tail conformation. The atomic coordinates for the complete EL homodimer molecular model is provided in **File S5 in Supporting Information S1**.

### Structural Analysis of EL Dimer with HSPG

#### A positively charged groove in the back of the EL dimer interacts with heparan sulfate proteoglycan

**Figure 7:**
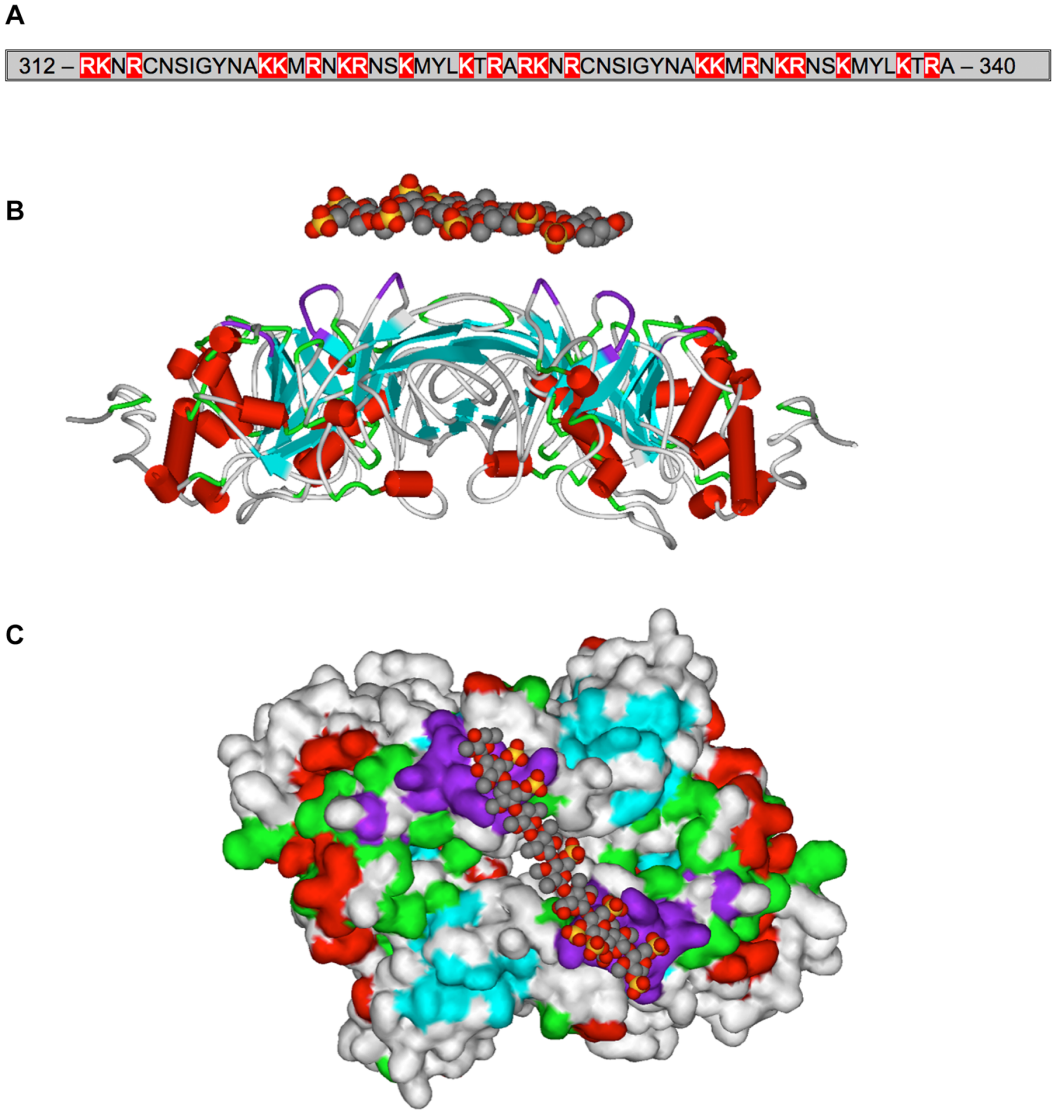
The back of EL structure contains a stretch of a sequence (312–340) with 10 Arginine (R) and 12 Lysine (K) and no negatively charged residues [Glutamic acid (E) or Aspartic acid (D)], which forms a positively charged groove, complementing negatively charged HSPG (A). Model of EL dimer with color-coded (purple) heparin binding sequence (**B**) and solvent accessible surface with docked decasaccharide (**C**). These structural studies predict how HSPG interacts with the EL homodimer.

Plasma lipases (LPL, HL, and EL) go through a complex maturation process in ER where monomers are assembled into fully functional homodimers [34]. All three lipases also have the ability to anchor as a homodimer to HSPG of endothelial cells, a property that is critical for their function. The model of the EL homodimer suggested a heparin-binding motif located in the back of the two monomers. This structural motif is within a stretch of 58 residues, of which 22 are positively charged, which forms a positively charged groove, complementing negatively charged HSPG (**Figure 7BC**). This stretch of the sequence (312–340) contains 10 Arginine (R) and 12 Lysine (K) but no negatively charged residues [Glutamic acid (E) or Aspartic acid (D)] (**Figure 7A**). Furthermore, our structural analyses showed the length of this heparin-binding motif is 75Å. The average length of a single molecule of a cyclic carbohydrate is 6Å. Thus, 11 units of cyclic carbohydrate are required to cover the basic amino acids in this motif. Measurement of the distance between the heparin-binding motif of two EL monomers revealed that the minimal number of cyclic carbohydrates is 5. The maximum is not defined due to the possible variety in secondary and tertiary structure of the oligosaccharide chain. In this model, a stretch of HSPG binds perpendicular to the intersection of EL homodimer.

### Structural Prediction of the EL-HDL Interaction

#### EL dimer has structural flexibility to accommodate any size HDL species

**Figure 8:**
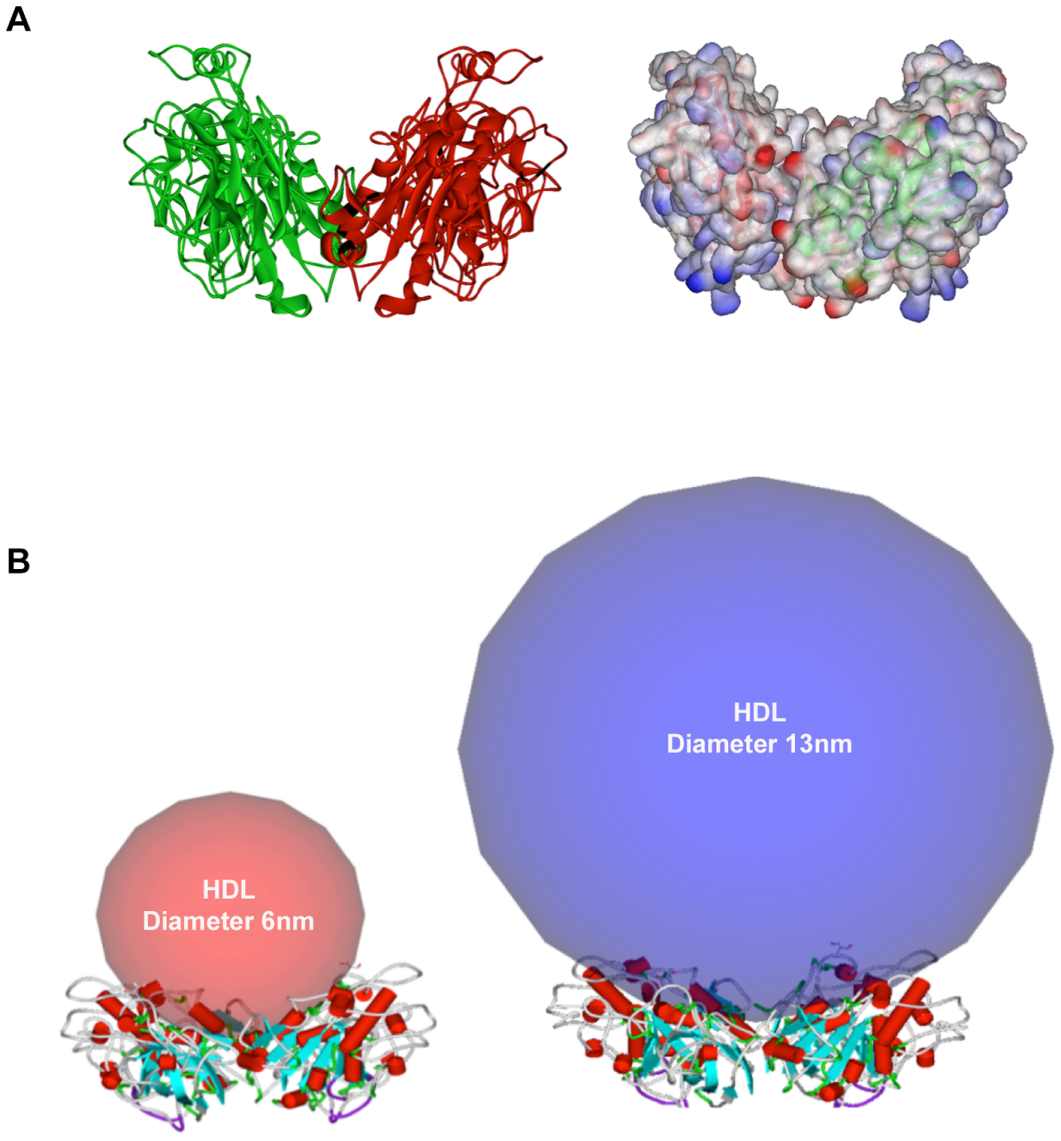
EL dimer interaction with HDL particles. EL dimer and its solvent accessible surface colored with electrostatic potential (**A**). The EL dimer is predicted to have the capacity and flexibility to accommodate different HDL particle sizes, ranging from 5.4 nm (Pre β-1) to 14.0 nm (Pre β-2) (**B**).

HDL particles are heterogeneous in size, ranging from 5.4 nm (pre β-1) to 14.0 nm (pre β-2) and are classified into five distinct subpopulations of decreasing size: HDL_2b_, HDL_2a_, HDL_3a_, HDL_3b_, and HDL_3c_ [4]. Our modeling studies suggest that when two molecules of EL form a homodimer, they create a wide “V” shape with a flexible hinge at the bottom (**Figure 8A**), which allows accommodation of different sphere sizes, including HDL_2_ and HDL_3_ particles (**Figure 8B**). This flexibility even allows larger sphere sizes such as VLDL, as experimentally shown for EL bridging VLDL, LDL, and HDL_3_ [15].

### Structure-Function Slope: A Novel Way to Assess Mutational Impact

#### There is a direct correlation between the impact a missense mutation has on EL structure net free energy and lipase function

**Figure 9:**
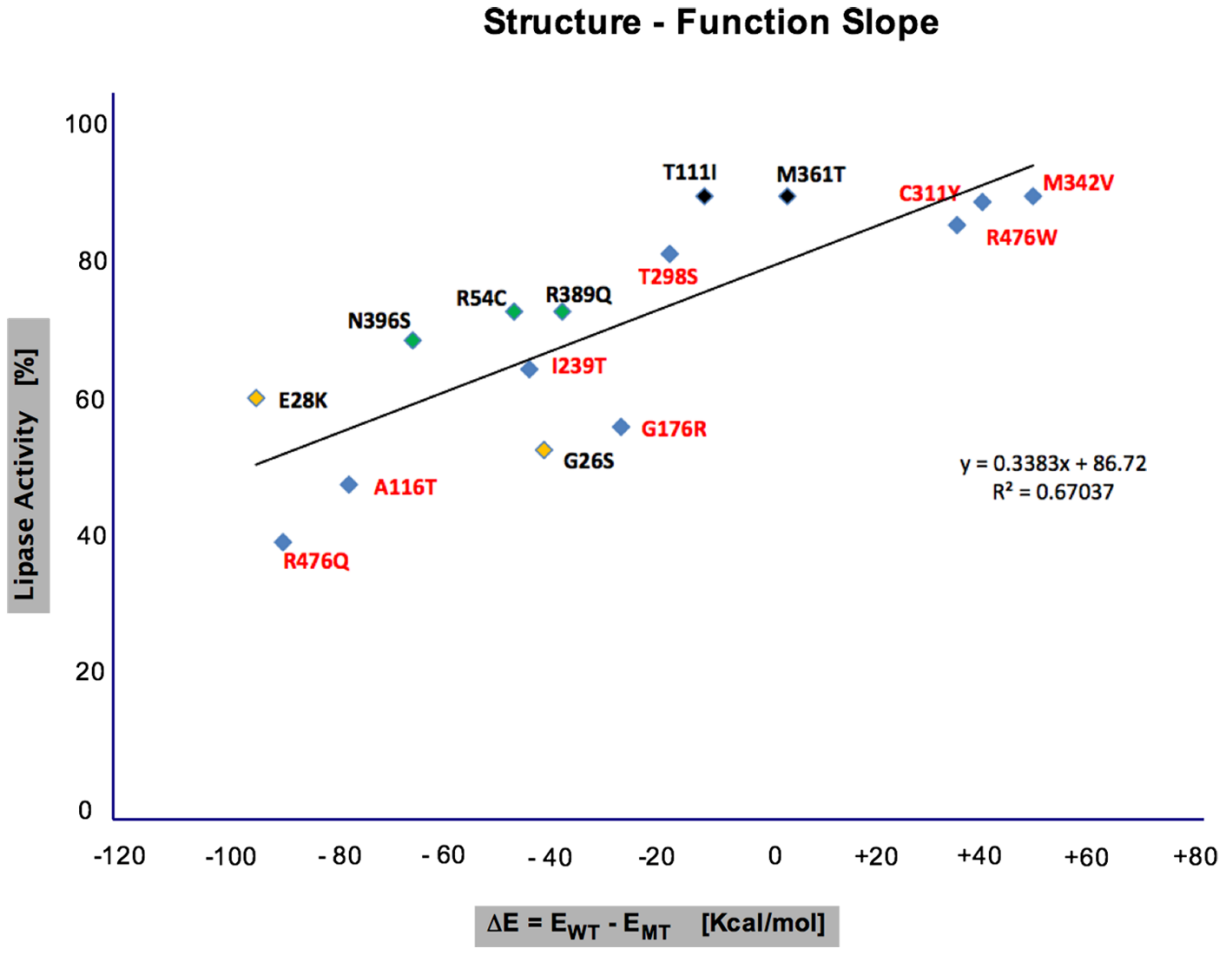
Impact prediction of missense mutations on EL structure and function using CHARMM energies. Mutants for which lipase activity of EL were experimentally determined are shown in black (100% lipase activity: T111I, M361T), green (80–90% lipase activity: R54C, R389Q, N396S), yellow (55–60% lipase activity: G26S, E28K), and the predicted lipase activity for other mutations in red.

Our 3D model of the human EL allowed us to analyze and to predict the potential impact of the known missense mutations on the structure and function of EL using their energy index. We hypothesized that there would be a direct correlation between the impact of any missense mutation on phospholipase activity of EL and the net difference in free energy between the wild type and mutant structures. Our structure-function analyses showed there was, indeed, such a correlation (**Figure 9**) (**File S3 in Supporting Information S1**). Using the experimentally determined phospholipase activity of wild type and ten mutant EL variants (**Figure 1**), we calculated the free energy difference between the structures with these missense mutations and the wild type EL structure, and used these results to generate a structure-function slope, which we used to predict the impact of the other mutations. Those mutations leading to total loss of lipase activity (N52S, P73L, T338P) were found to be outliers and were excluded from the slope calculation, which is predictable only for mutations leading to partial loss of lipase activity. The structure-function slope is a novel means of predicting the impact of a missense mutation on the function of a protein, and can be used in DNA and protein bioinformatics.

### Model-based Interpretation of the Impact of the Mutations on the EL Structure and Function

#### Structural examination of missense mutations provide insights about their impacts on EL structure and prediction on its function

It is also possible to predict the impact of most mutations on the structure by close structural examination of the location of the mutation. For instance, T338P is a substitution of hydrophobic proline for hydrophilic threonine. This residue is located in the hinge loop between the two domains. Furthermore, the threonine dihedral angles (φ = −93 and ψ = +176) cannot be adopted by the proline residue, thus this mutation leads to substantial conformational changes in both domains, which may impact both HDL binding and lipase activity of the enzyme. Such structural changes can be then correlated to our lipase assay and lipidomics data, which showed T3338P had little enzymatic activity. We have performed such analysis for all 18 missense mutations and detailed results are presented in **File S4 in Supporting Information S1**.

## Discussion

EL is a unique member of the lipase family because of its up-regulation by inflammatory cytokines, expression by endothelial cells, and extended PLAT domain, suggesting a special role for its phospholipase activity and bridging function in HDL metabolism and vascular health. In this respect, we sought to characterize EL genetically, functionally, structurally, and lipidomically.

### Genetic Association of the EL Missense Mutations

In a comprehensive resequencing of *LIPG* in subjects selected from the extreme ends of HDL-C distribution, we recently identified a number of non-exonic sequence variants associated with HDL-C levels [28]. In this study, however, our focus was on all known exonic variants in *LIPG*. In the literature, genetic associations of T111I with HDL-C levels or with other risk factors have been inconsistent. Some studies have found an association [18], [25], [35], [36], [37] while others did not [23], [24], [38], [39]. These discrepancies may stem from variations in population genetic structure. Our genetic screening for naturally occurring *LIPG* missense variants in the SLVDS biethnic population sample found only T111I to be a common variant (**Table 1**). Our structure-function studies of this EL variant showed that it had normal lipase activity with little impact on the structural integrity. However, our genetic association studies unexpectedly showed T111I had significant association with lower LDL-C and total cholesterol levels in our Hispanic population sample, but not in Whites (**Table 2**) (**File S1 in Supporting Information S1**). Interestingly, Durlach et al. [40] found an association with modest decreased in LDL-C levels in French diabetic patients with the T111I/TT genotype. We previously showed that the elusive association of T111I could be explained by its location within a haplotype block [28]. We found in Hispanics, T111I variant was part of the haplotype block 1 where causal variants may reside on the block; whereas, in Whites, it was located in a small but an independent region between blocks 3 and 4 where no significant SNPs reside. This not only explains the association pattern of T111I between Whites and Hispanics but also may provide an explanation for the discord among our genetic, functional, and structural studies of this well-known SNP. Based on our deep sequencing of *LIPG* gene [28], we identified a common haplotype (with frequency of 0.213) in Hispanics with three significant SNPs, which all were in linkage disequilibrium (LD) with one another. These three were T111I (in Exon 3), a novel SNP (rs61364338) in Intron 1, and another (rs34474737) in Exon1-UTR, suggesting the latter two are functional SNPs. Khetarpal et al. [41] recently showed rs34474737 decreased promoter activity using luciferase reporter assay and confirmed our finding that this variant was in strong LD with nonfunctional T111I. However, this does not explain the association of T111I with LDL-C and cholesterol in Hispanics. Our molecular modeling studies of T111I suggested this residue resides in dimer EL where HSPG binds. Therefore, T111I may impact the bridging function of the mutant EL, leading to enhanced LDL particle uptake and catabolism. Interestingly, in double EL/HL knockout mice, the combined deficiency of these two lipases resulted in accumulation of LDL, whereas in single knockout mice each was masked, suggesting that EL and HL play redundant roles in LDL catabolism [42]. Further studies will be required to fully elucidate the possible role of EL variant with T111I in LDL metabolism, including further confirmation of this novel genetic association in a larger cohort.

### Functional Analysis of the EL Mutations

Several missense mutations found in human *LIPG* have been functionally characterized, including G26S [20], T111I [19], [35], and N396S [19]. In a recent study, Edmondson et al. [19] showed that T111I had normal lipase activity and was not associated with HDL-C levels. In addition, they showed that the N396S mutation had little lipase activity and was associated with the high HDL-C group. This was the first published report of a loss-of-function missense mutation in *LIPG* that led to elevated HDL-C levels in humans. However, when we further analyzed their supplemental data, we noted that although the carriers of N396S (n = 23) had comparable and favorable lipid profiles (HDL, LDL, TC, TG) similar to the other high HDL-C group (n = 340), the carriers had lipoprotein (a) [Lp(a)] levels twice as high as the control group (mean = 73.6 with a SEM = 13.95 compared to a mean = 38.8 with a SEM = 2.02; p-value = 0.0137). Lp(a) is an important and independent CVD risk factor [43]. Although not discussed in the report, such significant elevation of Lp(a) raises major concerns about the potential inhibition of EL as a therapeutic method in raising HDL-C levels. Further research is required to show how lack of EL might influence Lp(a) levels. In a recent study by Voight et al. [44], *LIPG*-N396S was used in a Mendelian randomization approach to test if carriers of this mutation, by which raised their HDL-C levels, had reduced risk for myocardial infarction (MI). The investigators did not find N396S carriers had reduced risk MI and therefore questioned the atheroprotective properties of HDL. We noticed that their analyses again did not adjust for the elevated Lp(a) levels in the carriers. Interestingly, Hara et al. [45] tested whether the absence of EL is atheroprotective or atherogenic in a mouse model with deleted *LIPG*. The group found that lack of the EL enzyme leads to not only increases in HDL size and concentration, but also, surprisingly, to enhanced anti-inflammatory and anti-atherosclerotic functions of HDL. However, one should be cautious regarding extending the interpretation of this important animal study to humans, as there are major differences between HDL metabolism and function in mouse and humans, and hence mechanisms of atherosclerosis. G26S, which is also associated with high HDL-C levels, is an ethnic-specific rare missense mutation found predominantly in African-American populations. Functional studies showed this mutation has normal lipase activity but its secretion is significantly decreased, leading to increased HDL-C levels in the carriers [20]. The defect in secretion appears to be in the translation stage, and is not related to intracellular degradation. Interestingly, the group showed that the G26S mutant had enhanced bridging function, with a two-fold increase in binding lipoproteins (HDL and LDL) on the cell surface in comparison with the wild type. The significance of this unexpected enhancement in EL bridging function on CVD risk has also yet to be evaluated.

Our lipase activity measurements of G26S (39–44% reduced activity) and N396S (11–21% reduced activity) are different than those reported by Edmondson et al. [19] and Brown et al. [20]. The difference may be methodologic, as they used radioactive-labeled reconstituted HDL_3_; whereas, we used fluorescent-labeled micelles. However, similar to Edmondson et al. [19] and Smith et al. [35] we also found normal activity for T111I. In addition to the above missense mutations, we also report here for the first time on lipase activities of E28K, N52S, R54C, P73L, T338P, M361T, and R389Q. We found each had varying degrees of impact on EL phospholipase activity (**Figure 1BC**).

Unfortunately, we were unable to normalize the EL lipase activities to the amount of expressed EL, which we found was below the sensitivity level of detection by Western blot (**File S7 in Supporting Information S1**). However, there was little variability, between and within, in lipase activity of the EL wild type and mutants (**Figure 1B**). This indicates equal amounts of DNA may lead to little variability of expression. Moreover, the validity of this assumption is confirmed by Mitnaul et al. [68] who previously showed the level of EL protein expression correlates with its fluorescence lipase activity. However, the inability to normalize the activity of EL enzyme may have several caveats: It is not known if different mutations in the EL could lead to different rates of synthesis and to different intracellular trafficking of the protein. A significant portion of the mutant protein could also have been degraded in the cells before it reached the surface.

### Structural Analysis of the Mutations

Understanding the intrinsic ability of EL, and its isoforms, to interact with its substrate HDL, requires structural analysis of EL-HDL interactions. A two-domain structure with a hinge between and a lid covering the catalytic triad in the N-domain is a common structural theme among members of the lipase family (PL, LPL, HL, EL). However, we found the C-domain of EL contains a novel 23-aa α/β structural motif that gives its PLAT domain an edge, similar to a thumb (**Figure 6**). We do not know the functional significance of this element, but speculate that it may enhance the lipid binding function of the PLAT domain in a way that HL, which also binds to HDL, may not have. Homo-dimerization and binding to HSPG is critical for the lipase and bridging activities of LPL, HL, and EL. It has been shown that administration of heparin, which is closely related in structure to heparan sulfate, increases EL mass in plasma by 3 folds [80]. Our molecular modeling studies confirm the head-to-tail orientation of the EL dimer (**Figure 7**), as previously suggested for LPL [46] and EL [47]. Our molecular modeling studies also revealed that the dimerization allows for 4 patches of positively charged residues to come together to form a platform fit for interaction with negatively charged HSPG in a perpendicular manner. Such an EL-HSPG-EL complex allows the EL dimer to accommodate any HDL particle size with defined flexibility (**Figure 8**).

All 18 missense mutations (**Figure 5**) were subjected to modeling structural analyses (**File S4 in Supporting Information S1**) and structure-function correlation studies (**Figure 9**). We identified a direct correlation between the impact of any missense mutation on phospholipase activity of EL and the net difference in free energy between the wild type and mutant models (**Figure 9**). This graph can be useful in predicting the impact of almost any new mutation on EL function. The exceptions were that missense mutations leading to total loss of lipase activity, due to major structural conformational changes, have an unusual free energy index – well outside of the structure-function slope. Examples of such mutations are N52S, P73L and T338P. The excessive free energy index of these mutations may also lead to different heterodimer EL half-life in carriers of such mutations. A less flexible enzyme structure leads to increased enzyme half-life, as shown for N370S mutation in human acid β-glucosidase [48]. For other mutations with predicted reduced enzyme activity within the slope, and possible altered enzyme half-life, might have implications for HDL remodeling and inflammatory signaling pathways in the carriers.

### Lipidomics of EL-HDL Interaction

Gas chromatography/mass spectrometry (GC/MS) is a potent tool to analyze interaction between EL and native HDL particles. Our lipase assay results were supported by GC/MS analysis of the fatty acid patterns released from native total HDL incubated with either wild type or a mutant (T338P) EL (**Figure 2**), though the sensitivity of GC/MS is far superior to lipase assay. We found that EL does not completely hydrolyze phospholipids of HDL (**Figure 3**), even in the presence of high EL concentrations (**Figure 4**), suggesting that the role of EL is confined to conversion of HDL to smaller particles (HDL_2_ to HDL_3_) and not to complete HDL catabolism. This finding is in line with data by Jahangiri et al. [49] where they previously showed that EL remodels HDL and rHDL to smaller particles without the dissociation of apoA-I. The underlying mechanism for such observation can be speculated based on a regulatory mechanism shown for LPL enzymatic activity, which is down-regulated by high concentrations of generated fatty acids. In this form of regulation, LPL dislodges from its substrate (chylomicron or VLDL) and stops its lipolytic activities when concentration of the released free fatty acids around LPL reaches a critical level [81]. This is to prevent fatty acids from exceeding the capacity of tissues to take them up. Since LPL and EL are members of the same lipase family, a similar mechanism may exist for EL-HDL interactions as well.

Furthermore, our total HDL lipidomic analyses indicated that EL has a distinct and high preference for six fatty acids. These are palmitic acid (C16∶0), stearic acid (C18∶0), oleic acid (C18∶1), linoleic acid (C18∶2), arachidonic acid (C20∶4), and docosahexaenoic acid (C22∶6, DHA-omega 3) (**Figure 4**). Gauster et al. [13] also showed that EL had a similar preferential release of fatty acids from reconstituted HDL_3_. The polar head group of a phospholipid at least in part determines EL preference [50]. Such a differential preference has profound implications for lipid-based signaling pathways in inflammation.

The fatty acid released by EL at the highest rate is palmitic acid (PA, C16∶0), which is essential for protein palmitoylation. This is a process by which palmitate is attached covalently to select cysteine residues of a protein via a thioester linkage and so-called S-palmitoylation. This post-translational modification has diverse effects on protein function and localization, and has been discussed in several recent reviews [51], [52]. In addition, it has been shown that production of the inflammatory cytokine TNFα in macrophages is markedly increased by palmitic acid [53]. Interestingly, palmitic acid, along with myristic acid and palmitoleic acid, promotes physiological heart growth via signal pathways in both python and mouse [54].

The next most rapidly liberated fatty acid from HDL is linoleic acid (LA, 18: 2n−6) along with its derivative, alpha linolenic acid (ALA, 18: 3n−3), which are omega-6 (ω6) and omega-3 (ω3) polyunsaturated fatty acids (PUFAs), respectively. LA is a precursor for arachidonic acid (AA, 20: 4n−6) and ALA is a precursor for eicosapentaenoic acid (EPA, 20: 5n−3) and docosahexaenoic acid (DHA, 22: 6n−3). AA and EPA, in turn, are precursors for different classes of pro- and anti-inflammatory signaling molecules, respectively [55]. Furthermore, arachidonic acid itself inhibits gene expression of adhesion molecules (E-selectin, ICAM-1, IL-8) in activated endothelial cells [56].

PUFAs also have an important role in modulating gene expression by regulating several nuclear transcription factors, including PPARs, LXRs, and SREBP-1c, which in turn, play crucial roles in the regulation of genes involved in lipid metabolism and inflammation [57], [58]. When in the cell membrane, PUFAs also contribute to its fluidity, which is an important factor for correct hormone-receptor binding. For example, an insulin receptor cannot embed in a rigid membrane. In addition, when intercalated in the cell membrane, PUFAs and their metabolites exert a second messenger action (eicosanoid signaling molecules) [59].

Released fatty acids by EL have dual roles in inflammation: Ahmed et al. [60] demonstrated that HDL-mediated repression of leukocyte adhesion to endothelial cells depends on the lipase activity of EL, which leads to PPARα activation and subsequent VCAM1 repression. VCAM1 is a cell adhesion molecule that mediates adhesion of leukocytes to vascular endothelium, leading to vascular inflammation, one of the early stages in atherosclerosis development. On the other hand, Riederer et al. [61] showed the pro-inflammatory side of EL lipase activity. Interleukin 8 (IL-8) is a pro-inflammatory chemokine and is implicated in the pathogenesis of atherosclerosis. Its expression by endothelial cells is induced by EL-generated lipolytic products (LPC 16∶0, 18∶1, 18∶2, 20∶4) from HDL. Interestingly, IL-8 induction by EL has different potencies depending on the acyl-chain length and degree of saturation of liberated fatty acids.

Altogether, these studies indicated that the released free fatty acids are lipid mediators, which carry potent pro- and anti-inflammatory properties. Thus, it is reasonable to speculate that HDL carries fatty acids that also contribute to the atheroprotective properties of HDL, and contribute to immunological functions, further implicating a critical role for EL in vascular inflammatory processes and conditions. Quantitative-longitudinal studies will be required to elucidate the impact of release of these preferred FFAs on signaling pathways.

**Figure 10:**
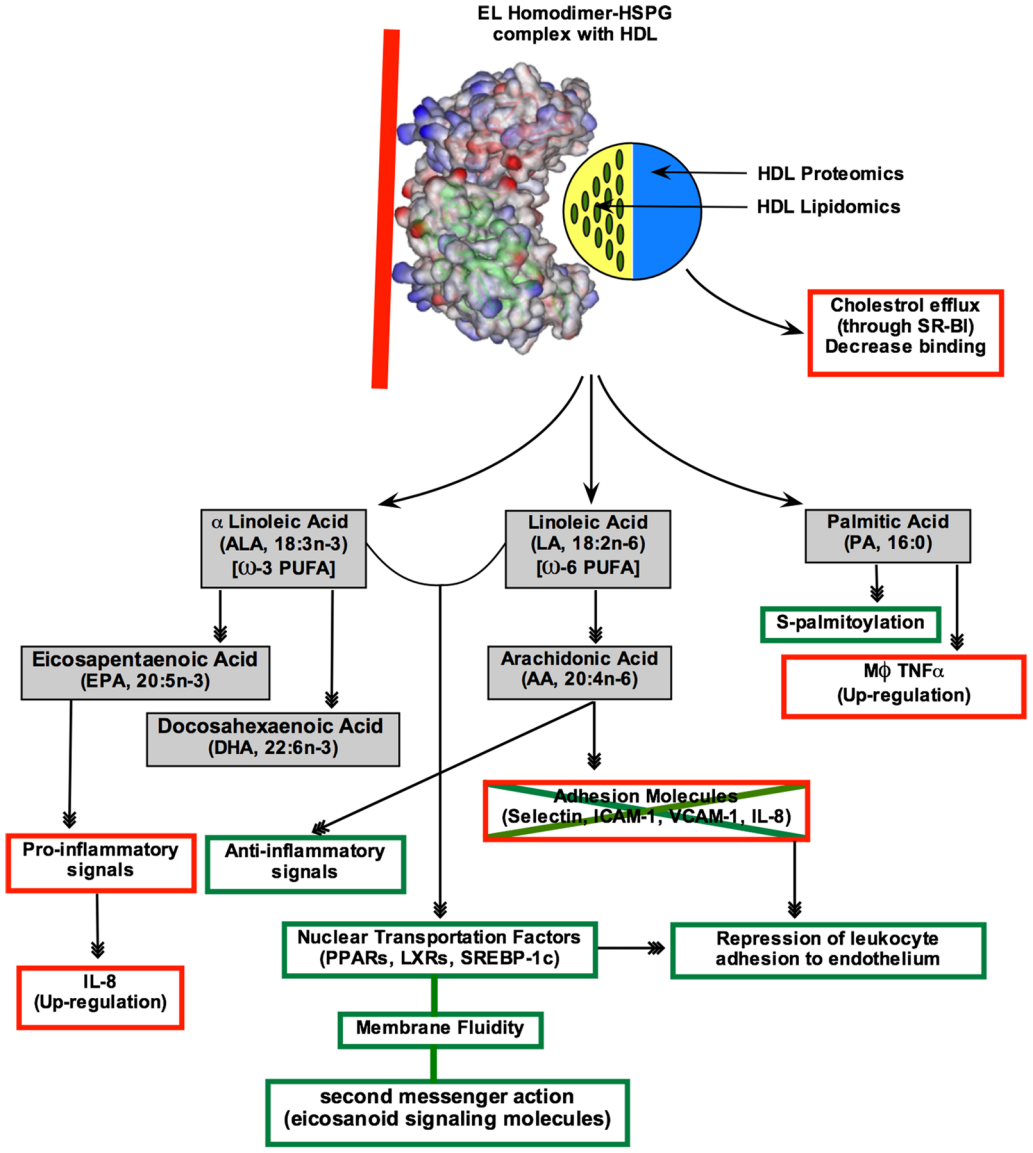
The schematic diagram depicting the dual role of EL in vascular homeostasis by remodeling HDL lipidomics. The red and green boxes indicate the cascades leading to pro- and anti-inflammatory pathways.

Finally, atherosclerosis is an inflammatory disease, in which EL has a dual role (**Figure 10**). The critical question regarding the role of EL in vascular health, therefore, is to determine how the HDL-released free fatty acids choose activation or inactivation of signaling pathways involved in inflammation. Concentrations of these FFAs obviously play a major role in modulating the signal intensities of inflammatory pathways. If FFA concentration would also determine signaling pathway selection, then the levels of EL lipase activity would play a critical role in the determination which pathways (pro- or anti-inflammation) are activated. Functional analysis of inflammation in subjects with various variants of EL (i.e., with 100%, 50%, or 0% lipase activity) should shed light on the role of EL in atherosclerosis. Furthermore, another critical factor is HDL-C levels. In subjects with low HDL-C levels, there would be less substrate for EL to act on, resulting in a reduction of FFA levels and hence lower concentration of the lipid mediators. This may lead to up-regulation of inflammation pathways and susceptibility to atherosclerosis.

In summary, EL missense variants seen in human population studies were subjected to genetic, structural, functional, and lipidomic analyses. The results provide novel insights into EL biology that should serve as a platform for dissecting the role of this fascinating lipase in vascular health.

## Materials and Methods

### Population

The San Luis Valley Diabetes Study (SLVDS), which was initiated in the mid-1980's, was a case-control study of Type 2 diabetes in a biethnic population of non-Hispanic Whites (Whites) and Hispanics in southern Colorado. The design and objectives of the population study and methods of measuring various biometrics have been described in detail [62]. The cardiovascular risk factors [63] and the quantitative traits related to lipoprotein metabolism [64] of these two populations have previously been assessed. The suitability of the San Luis Valley population arises from its stability based on historical demographic records, similar environmental conditions, and different genetic backgrounds with little genetic admixture between the two sub-populations [65]. The entire SLVDS population samples (n = 1791) consisted of 659 diabetic cases and 1132 normoglycemic (preprandial normal blood glucose level of 90–110 mg/dl) controls. The non-diabetic control sample, which was used for the genetic association portion of this study, included 674 Whites and 458 Hispanics. However, at the time of genetic screening of the control samples, we had available 426 samples for Whites and 376 for Hispanics. The age range of study participants was 20 to 74 years. Written informed consent from the SLVDS participants was previously obtained and the IRB protocol for this study was approved by the Colorado Multiple Institutional Review Board.

### Functional Analyses

#### Site-directed Mutagenesis and Cloning

The EL mutants were constructed using the overlap extension PCR method [66]. In brief, primers were made in both the sense and anti-sense orientation at the site of the desired mutation. The single nucleotide change was introduced into both the sense and anti-sense strands. PCR was performed pairing a primer encompassing the first 16 nucleotides of the coding sequence of *LIPG* with the addition of the Kozak consensus sequence [67] (*LIPG* 5′ primer Kozak: GCCACCATGAGCAACTCCGTTC) with an anti-sense oligo containing the mutated nucleotide. Concurrently, another PCR was performed pairing a sense oligo containing the mutation with an antisense oligo that encoded the final 23 nucleotides of the *LIPG* coding sequence ending with the three nucleotides that form the termination codon (*LIPG* anti TGA: TCAGGGAAGCTCCACAGTGGGAC). The list of the primers used in the study is provided in **File S6 in Supporting Information S1**. The two PCR fragments were combined in a ligation reaction that joined them into the full-length gene containing the single nucleotide change corresponding to the desired EL mutant. This fragment was then cloned into the pCDNA3.1 TOPO vector (Invitrogen, Cat # K480001) and transformed into ONESHOT competent cells according to the manufacturer's protocol (Invitrogen, Cat # C404003). Positive colonies were picked and grown, and miniprep DNA was obtained using a Zymogen Zippy DNA purification miniprep kit (Zymogen, Cat # D4036). The resulting DNA was sequenced to confirm that both the desired mutation was present and that there were no spurious mutations. Once a colony was identified as having the desired mutation, it was grown in larger quantity, and highly purified DNA for transfection was prepared using an endotoxin-free Maxiprep Kit (Qiagen, Cat #12362).

#### Cell Culture

HEK-293 cells were used for transfection. The cells were grown in DMEM (Invitrogen, Cat# 12430104) with 10% calf serum (Invitrogen, Cat# 16170060) and a 100-fold dilution of Penicillin (100 units/ml)/Streptomycin (100 μg/ml) (Invitrogen, Cat# 15140122). The cells were split just before reaching 100% confluency when passaging for maintenance.

#### Transfection

FUGENE transfection kit (Roche, Cat# 1814443) was used to introduce the *LIPG* mutants into the HEK-293 cells, following the protocol provided by the manufacturer. In brief, we used both 24-well and 96-well plates and plated the recommended 50,000 and 10,000 cells per well, respectively. We used the recommended volumes of FUGENE reagent, and Opti-MEM media (Invitrogen, Cat # 31985) along with the recommended quantity of DNA to transfect either 24-well or 96-well plates. For 24-well plates, we mixed 0.6 µl of FUGENE reagent per well and 0.2 µg DNA per well into 500 µl Opti-MEM per well. For 96-well plates, we used 0.15 µl FUGENE and 0.005 µg DNA mixed in 100 µl Opti-MEM. In order to minimize well-to-well pipetting errors, all regents for a series of transfections with the same plasmid were pooled into one master mix. First, FUGENE was mixed with Opti-MEM and incubated at room temperature for 5 minutes. Then, an appropriate volume of plasmid DNA was added to yield the quantity of DNA called for, and incubated for an additional 30 minutes at room temperature. The mix was added to the appropriate well and the transfected cells were allowed to express the EL protein for 24 hours, at which time the lipase assay was performed. In addition, each transfection was repeated 3 times and performed in triplicate in order to average potential variation in expression between and within transfections. Although to normalize enzyme activity to protein mass is standard procedure, this was not possible in our lipase assays as the level of EL expression was consistently below the level of Western's blot sensitivity (**File S7 in Supporting Information S1**). To compensate for this drawback, the expression protocol was kept identical for the WT EL and the mutants, which led to little and consistent variations in lipase activity (**Figure 1B**).

#### Protein Immunoblot

Western blot was used to detect mature EL protein in transfected HEK-293 cells media. Following transfection, cells were washed with 1× PBS (with no Ca or Mg). Opti-MEM serum free medium (Invitrogen, Cat # 31985), with 10 u heparin per ml (to release EL from HSPG of the cell membrane to the media) was added to each well (1.5 ml/well) and incubated for 30 min. Media was collected and centrifuged at 1000 rpm for 5 min. This media was used as a source of EL activity (for lipase assay, mass spectroscopy and Western blot) and stored at −80°C with 15% glycerol final concentration. Rabbit polyclonal anti-EL antibody (Novus Biologicals, Cat# NB400-111) was used in the protocol provided by the manufacturer for both Coomassie-stained SDS-PAGE and Western blot gels (**File S7 in Supporting Information S1**).

#### Fluorogenic Micelle Substrate

We used fluorogenic micelle substrates for the lipase assay, as they were previously shown to be a suitable substrate for EL [68]. A solution of 0.5 mg/ml bis-BODIPY-PC fluorescent phospholipids (**Figure 1A**) (Invitrogen, Cat# B7701) dissolved in ethanol was prepared (100 μg dissolved in 200 µl ethanol) along with a solution of 0.2% triton X-100 dissolved in chloroform (20 µl in 10 ml). Equal volumes of both solutions were mixed (to final triton concentrations of 0.1%), vortexed, and dried to completion under argon gas to avoid oxidation. The triton formed the bulk of the micelles containing the BODIPY-labeled phospholipids. The dried micelles were re-suspended in 970 µl PBS and mixed well to the final concentration of 100 μM (A 100X solution). The resulting labeled substrate was stable for at least 4 months at 4°C.

#### Lipase Assay

The majority of the lipase assays were performed in 24-well plates. After 24 hours of transfection, the cells were rinsed once with serum-free media. More rinses would risk disturbing the cell monolayer. The micelle substrate was diluted 100x in serum-free media and 200 µl of substrate/media was added to each well and incubated for one hour. After the incubation, the supernatant (200 µl) was carefully removed from the cells and transferred to a black-coated 96-well plate with clear bottom. The EL enzyme is attached to heparan sulfate proteoglycan on the surface of the cells and so is not transferred to the plate. The plate was read in a fluorescent plate reader (20/20^n^ Luminometer, Turner Biosystems) and the total fluorescence in each well was quantified.

#### Lipase Data Analysis

The plate reader program provided the mean and standard deviation for the collect data and analysis of variance (ANOVA) was performed using Microsoft Excel.

#### Pyrosequencing

We used pyrosequencing, which represents an accurate, robust, and high throughput method for SNP analysis to screen SLVDS population samples for selected *LIPG* missense mutations. Biotinylated PCR products targeting each missense mutation were immobilized on streptavidin-coated Dynabeads (Dynal AS, Oslo, Norway) according to a standard protocol by incubating 125 µg Dynabeads with 5 pmoles PCR products for each SNP for 30 minutes at 43°C. The Dynabeads were transferred to a PSQTM 96 plate, and after washing, the products were incubated in 20 µl 0.1 M NaOH for 5 minutes to obtain single-stranded DNA (ss-DNA). The immobilized strand was then washed and annealed with 1 pmole sequencing primer in 40 µl annealing buffer at 95°C for 1 minute, 65°C for 1 minute, with subsequent cooling to room temperature. Real-time pyrosequencing was performed at 28°C in a total volume of 50 µl in our core-facility automated 96-well PSQ pyrosequencer using PSQ 96TM SNP reagents (Pyrosequencing AB, Uppsala, Sweden) and the PSQ 96 plate containing the magnetic beads with the primer annealed to immobilized ss-DNA. After completion, base calling of the pyrograms, pattern recognition, and assignment into amino acid sequences was performed automatically and confirmed by manual inspection.

### Genetic Analyses

#### Genetic Association

Allelic and genotypic frequencies of the screened missense mutations were determined by counting. Multiple linear regression was used for genotype-phenotype association studies. The analyses were performed separately for Whites and Hispanics. The following covariates were considered for the regression model: gender, age, body mass index (BMI), and smoking. In order to achieve a more normal distribution, the following traits were subjected to natural log (NL) prior to being included in the regression model: HDL (total), HDL_2_, cholesterol, triglyceride, and BMI. Stepwise variable selection was performed first to determine significant covariate(s) for inclusion in the genotype-phenotype association models (with testing threshold of 0.05). Next, the effect of the T111I SNP was added. The effect of T111I on the lipid panel measurements was tested using both a genotypic and allelic parameterization. For allelic association, the genotypes were recorded, as G, in order to give weight to each genotype as follows: CC  = 0, CT  = 1, TT  = 2. This led to the allelic association model:
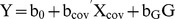
where the b_cov_′X_cov_ allows for adjustment of covariates deemed significant by the variable selection procedure. For genotypic association, two dummy variables were created and included in the regression model as follows: D_TT = 0 (for T/C and C/C genotypes); D_TT = 1 (for T/T genotype); D_TC = 0 (for T/T and C/C genotypes); D_TC = 1 (for T/C genotype). This led to the genotypic association model:



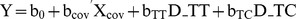



Similarly, dummy variables were created for the covariate ‘smoking’: D_Smoker = 0 (for nonsmokers and ex-smokers) and D_Smoker = 1 (for current smokers). Regression coefficients, which indicate the amount by which the tested variable is changed when a covariate increases by one unit, are denoted by “B-value”, and such estimates are provided.

### EL Molecular Modeling

#### Homology Modeling of EL

3D molecular structure of EL is not known yet. So, we used homology-modeling techniques to generate a 3D molecular model of EL. EL is a 500-residue protein, including a 20-residue signal peptide; the mature enzyme is 480-residues long and is glycosylated (**File S2 in Supporting Information S1**). The referenced residue numbering here is based on the full length of 500 residues. In brief, ten pancreatic lipase (PL) structures for which the two states of PL catalytic site (open and closed lid – PDB # 2PVS and 1LPA, respectively) have been captured by X-ray crystallography were used as templates [69], [70]. The open-lid state of the lipases is an active state in which a catalytic reaction can occur. We built two models, one based on the open lid and the other on the closed lid using Accelrys Discovery Studio Modeler [71], but used only the open-lid model for the subsequent structural analysis. We selected three PDB structures of PL as templates: 2PVS (chains A and B) and the one-chain structure of 1LPA. We separated the two chains in the 2PVS structure and treated them as two independent templates. The Verify Protein (Modeler) protocol allows the selection of the best structure from a collection of protein molecules with the same protein sequence. This protocol uses the Modeler DOPE (Discrete Optimized Protein Energy) method [72] to calculate a DOPE score for each structure. A lower score indicates a statistically better model. The models were subjected to energy minimization using the Accelrys CHARMm protocol in Discovery Studio in order to obtain refined structures. The 3D models were finally tested for geometric accuracy (chirality, peptide bond geometry and stereochemistry, and missing residues) using Protein Check (Discovery Studio, Accelrys).

#### Novel Structural Motif

The C-domain of each of the three lipases (LPL, HL, EL) forms a PLAT domain, which is involved in lipid interaction. The PLAT domain (347–482 aa) in EL differs from the other lipases by the insertion of a 23-aa loop (418 – GASQSWYNLWKEFRSYLSQPRNPG – 441). A search for homologous PDB structures by sequence similarity to this loop yielded no structures, indicating this loop may adopt a novel structure. The alternative means of identifying a 3D template for a given protein sequence is to compare its sequence secondary structure and its profile (fold) to the sequences of proteins with experimentally determined 3D structures. In order to find a template structure for this loop, we used the online threading tool Phyre [73]. Phyre can generate a 3D model from predictions based on consensus secondary structures derived from 3D structures in PDB. Both profile and secondary structure are then used as a query to scan the fold library using a profile-profile alignment (PPA) algorithm [74]. This alignment process returns a score upon which the alignments are ranked and identifies the 3D template structure. For this 23-residue loop, the Phyre server predicted a high helical content, which is consistent with the profile of a coil/helix element found in the x-ray crystal structure of RNA polymerase from Archaea (PDB# 3HKZ), [75]. This structure was the only one identified with as much as 30% sequence homology. An additional criterion in selection of the template for the loop was the distance between Cα atoms on the two conserved amino acids preceding the loop (Glu^417^ and ending the loop Arg^442^) in comparison with other lipases. This distance in the crystal structure of available lipases was 15Å on average – the same distance was also observed in the 3HKZ structure. In preparation for homology modeling the structures of 2PVSA, 2PVSB, and 1LPA were first aligned by multiple sequence alignment; then the structure representing the EL loop 418–441 was modeled into these structures. This composition of three lipases and a small portion of 3HKZ representing an additional loop were aligned with the sequence of EL and subjected into homology modeling using the Accelrys Modeler suite. Ten homology models were ranked, and the lowest scoring model was selected for the further analysis.

#### Mutant Design

All 18 mutants were substituted in the EL model using Accelrys DS protocol Build Mutants [76]. This protocol substitutes selected residues to specified types and optimizes the conformation of both the mutated residues and any surrounding residues that lie within a specified cutoff radius, in our case 3.5Å. The last step of the protocol, which calculates the DOPE scores for each mutant model, reports the scores in the output for evaluating the quality of the models. In order to be able to compare the energy between the wild type and mutant structures both accurately and appropriately, we calculated energy for each of the mutants equivalent to wild type using the same protocol, by optimizing surrounding amino acids within a 3.5Å radius. By this means, it was ensured that the optimization was done the same way for each mutant, and each was related to wild type.

#### CHARMM Minimization

The final homology model of the wild type EL with its novel loop was minimized using CHARMM minimization protocol (www.charmm.org) in Accelrys Discover Studio. Subsequently, CHARMM minimizations were applied to all generated mutants and their corresponding wild types. All calculations were run in gas phase and in the solvent using Generalized Born (GB) implicit solvent model [77]. These calculations were used to evaluate the relative stability of mutants and to derive free energy estimates for each of them:
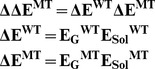
Where ΔΔΕ^MT^: Free energy value for the mutant, ΔΕ^WT^: Energy difference for wild type (WT), ΔΕ^MT^: Energy difference for the mutant (MT), E_G_: Energy in gas phase, and E_SOL_: Energy in solvent phase.

#### Mutational Structure-Function Analyses

Each mutant structure created *in silico* was mutated back to its wild type using the same protocol. This was done in order to ensure the same optimization level for the selected mutant/wild type and its surrounding. As described above, all mutants and corresponding wild types were subjected to the CHARMM calculations. We then calculated the free energy difference between these missense mutations and the wild type EL structure, leading to a structure-function slope that can be used for the estimation of new mutation impact on EL activity. All the calculations were carried out using the GB implicit solvent model.

#### EL-HDL Structural Interaction Analyses

The homodimer structure of EL was minimized using CHARMM protocols in order to prevent intra- and inter-molecular clashes as well as to ensure best side-chain configurations. This was followed by calculations of solvent accessible surfaces, then colored by the Delphi calculated electrostatic potentials [78]. In order to mimic HDL particles, different sizes of spherical particles were created and docked into the site. The sizes were selected to mimic the smallest and the largest measured HDL particle sizes. Docking of the spherical particles into the homodimer site was done using the ZDOCK program [79]. The Dock Proteins protocol provides rigid body docking of two protein structures using the ZDOCK algorithm as well as clustering the poses according to the ligand position, which allowed preserving non-bonded distances between the homodimer solvent accessible surface and the particle surface.

### HDL-EL Lipidomics Analyses

#### HDL Preparation

Purified human total HDL (d = 1.063–1.21 g/ml) was obtained commercially (Kalen Biomedical, cat # 770300). According to the manufacturer's records, the total HDL was from a male donor with normal lipid panel, including HDL-C of 68 mg/dl.

#### Gas Chromatography/Mass Spectrometry Sample Preparation

To determine the amount of released fatty acids from human total HDL by the wild type and one mutant EL with little lipase activity (T338P), different amounts of both cell media (40, 80, and 150 µl) were added separately into 40 µl of 1 mg/ml HDL. The mixtures of HDL with wild type as well as mutant were incubated at 37°C for 1 hour in a heat block (VWR Scientific). As a negative control, we used 40 µl of untreated 1 mg/ml HDL (without adding wild type or mutant media). Heptadecanoic acid (C17∶0) (10 µg/ml dissolved in n-heptane) was used as an internal standard (ISTD). After extraction of samples with n-hexane, the organic phase was evaporated to dryness under nitrogen flow. Subsequently, 1 ml methanolic acid (4 ml methanol + 1 ml 12 M HCl) was added to the residue, and the tube was closed with the screw cap. The tube was then heated at 120°C for 45 minutes. After cooling the sample, 2 ml of a mixture of n-hexane: water (v:v 1∶1) was added into the tube and vortexed briefly, and centrifuged until both layers were clear. The organic phase was transferred to a new centrifuge tube and dried under nitrogen. 200 µl of n-hexane was added to the residue and after vortexing, the sample was transferred to an HPLC vial containing a 200 µl insert. The samples were analyzed by gas chromatography/mass spectrometry (GC/MS).

To assess the HDL fatty acid composition, 100 µl of 1 mg/ml HDL spiked with 10 µg/ml heptadecanoic acid (C17∶0) as internal standard. The mixture was incubated at 120°C in a heat block for 2 hours. All other steps including extraction and derivatization were performed under the same conditions as described above for incubation of the wild type or mutant EL with HDL.

#### Gas Chromatography/Mass Spectrometry Procedure

Separation and quantification of released fatty acids was carried out using an Agilent Technologies 6890N gas chromatograph coupled to an Agilent Technologies 5973 quadrupole mass selective detector. A DB-23 (Agilent Technologies) capillary column (30 m×0.25 mm inner diameter, 0.25 µm film thickness) was used for separation of fatty acids. The temperature of the injector was 270°C, and the sample (1 µl) was injected in the splitless mode. The column temperature was set to 140°C, and ramped in three stages: at a rate of 15°C/min to 170°C, at a rate of 5°C/min 170°C to 200°C, and at a rate of 30°C/min 200°C to 240°C. The temperature was held for 5 minutes at 240°C. The quadrupole mass spectrometer was operated in the electron impact scan mode.

## Supporting Information

Supporting Information S1 **File S1**, The lipid panel in the SLVDS carriers of *LIPG* T111I missense. **File S2**, Human EL protein sequence with highlighting all known structural motifs and missense mutations. **File S3**, Structure-function correlation of all known missense mutations in EL. **File S4**, Structural close-up of all known missense mutations in EL structural model. **File S5**, Atomic coordinates for the complete EL homodimer molecular model (separate file, PDB format). **File S6**, The list of primers used in *LIPG* mutagenesis. **File S7**, Western blot of media containing EL.
(ZIP)
