# Supplementary material for: Genetic and Structure-Function Studies of Missense Mutations in Human Endothelial Lipase
Source: PLoS One. 2013 Mar 25;8(3):e55716. doi: 10.1371/journal.pone.0055716 (PMC3607615; doi:10.1371/journal.pone.0055716)
Supplement: Supporting Information S1 — File S1, The lipid panel in the SLVDS carriers of LIPG T111I missense. File S2, Human EL protein sequence with highlighting all known structural motifs and missense mutations. File S3, Structure-function correlation of all known missense mutations in EL. File S4, Structural close-up of all known missense mutations in EL structural model. File S5, Atomic coordinates for the complete EL homodimer molecular model (separate file, PDB format). File S6, The list of primers used in LIPG mutagenesis. File S7, Western blot of media containing EL. (ZIP) [file pone.0055716.s001.zip › Supporting Files/S-1.pdf]

S – 1. Lipid panel of the *LIPG* T111I genotypes in the bi-ethnic population samples of San Luis Valley in Colorado.

		Whites				Hispanics			
Lipid Panel	T111I	N	Mean	SEM	SD	N	Mean	SEM	SD
CHOLESTEROL	**CC**	223	218.23	3.183	± 47.534	224	209.08	2.870	± 42.948
	**TC**	171	214.96	3.093	± 40.451	136	216.94	3.876	± 45.207
	**TT**	32	208.47	6.121	± 34.623	16	239.62	9.003	± 36.013
	Total	426	216.19	2.128	± 43.926	376	213.23	2.265	± 43.926
TRIGLYCERIDE	**CC**	223	138.91	7.465	± 111.470	224	148.16	4.620	± 69.139
	**TC**	171	149.63	5.930	± 77.543	136	151.38	6.241	± 72.785
	**TT**	32	157.16	28.869	± 163.306	16	146.81	19.548	± 78.190
	Total	426	144.58	5.054	± 104.307	376	149.27	3.646	± 70.689
TOTAL HDL	**CC**	223	51.85	1.043	± 15.577	224	48.49	0.899	± 13.454
	**TC**	171	50.30	1.241	± 16.233	136	49.08	1.300	± 15.166
	**TT**	32	51.69	2.804	± 15.859	16	53.88	3.277	± 13.109
	Total	426	51.21	0.768	15.845	376	48.93	0.727	± 14.088
HDL_2	**CC**	223	10.32	0.563	± 8.406	224	8.40	0.387	± 5.794
	**TC**	171	9.83	0.650	± 8.502	136	8.57	0.683	± 7.964
	**TT**	32	9.81	1.578	± 8.924	16	10.06	1.253	± 5.013
	Total	426	10.08	0.410	± 8.467	376	8.53	0.342	± 6.626
HDL_3	**CC**	223	41.39	0.659	± 9.836	224	40.09	0.626	± 9.376
	**TC**	171	40.47	0.731	± 9.553	136	40.51	0.813	± 9.485
	**TT**	32	41.88	1.721	± 9.734	16	43.81	2.321	± 9.282
	Total	426	41.06	0.470	± 9.706	376	40.40	0.486	± 9.417
LDL	**CC**	223	137.89	2.965	± 44.282	224	130.48	2.716	± 40.647
	**TC**	171	134.44	2.871	± 37.544	136	136.28	3.788	± 44.172
	**TT**	32	124.62	6.457	± 36.525	16	156.39	9.247	± 36.988
	Total	426	135.51	1.996	± 41.204	376	133.68	2.170	± 42.074
