# Supplementary material for: Genetic and Structure-Function Studies of Missense Mutations in Human Endothelial Lipase
Source: PLoS One. 2013 Mar 25;8(3):e55716. doi: 10.1371/journal.pone.0055716 (PMC3607615; doi:10.1371/journal.pone.0055716)
Supplement: Supporting Information S1 — File S1, The lipid panel in the SLVDS carriers of LIPG T111I missense. File S2, Human EL protein sequence with highlighting all known structural motifs and missense mutations. File S3, Structure-function correlation of all known missense mutations in EL. File S4, Structural close-up of all known missense mutations in EL structural model. File S5, Atomic coordinates for the complete EL homodimer molecular model (separate file, PDB format). File S6, The list of primers used in LIPG mutagenesis. File S7, Western blot of media containing EL. (ZIP) [file pone.0055716.s001.zip › Supporting Files/S-2.pdf]

S – 2. Annotated human EL primary protein sequence with the location of the structural motifs and known to-date missense mutations.

```
        10         20         30         40         50         60
MSNSVPLLCF WSLCYCFAAG SPVPFGPEGR LEDKLHKPKA TQTEVKPSVR FNLRTSKDPE
          Signal Peptide

        70         80         90        100        110        120
HEGCYLSVGH SQPLEDCSFN MTAKTFFIIH GWTMSGIFEN WLHKLVSALH TREKDANVVV

       130        140        150        160        170        180
VDWLPLAHQL YTDAVNNTRV VGHSIARMLD WLQEKDDFSL GNVHLIGYSL GAHVAGYAGN

       190        200        210        220        230        240
FVKGTVGRIT GLDPAGPMFE GADIHKRLSP DDADFVDVLH TYTRSFGLSI GIQMPVGHID

       250        260        270        280        290        300
IYPNGGDFQP GCGLNDVLGS IAYGTITEVV KCEHERAVHL FVDSLVNQDK PSFAFQCTDS
                          Lid

       310        320        330        340        350        360
NRFKKGICLS CRKNRCNSIG YNAKKMRNKR NSKMYLKTRA GMPFRVYHYQ MKIHVFSYKN
                                Heparin binding

       370        380        390        400        410        420
MGEIEPTFYV TLYGTNADSQ TLPLEIVERI EQNATNTFLV YTEEDLGDLL KIQLTWEGAS

       430        440        450        460        470        480
QSWYNLWKEF RSYLSQPRNP GRELNIRRIR VKSGETQRKL TFCTEDPENT SISPGRELWF

       490        500
RKCRDGWRMK NETSPTVELP
```

Signal Peptide:	20aa (1-20)
Active Site:	S[169], D[193], H[274]
Disulfide Bonds:	S[64] – S[77], S[252] – S[272], S[297] – S[316], S[308] – S[311], S[463] – S[483]
Glycosylation Sites:	N[80], N[136], N[396], N[469], N[491]
Heparin binding:	13aa (325-337)
PLAT domain:	136aa (347-482)
_ Lid structure:	21aa (252-272)

Missense Mutations (To-date): G26S, E28K, N52S, R54C, P73L, T111I, A116T, G176R, I239T, R276ter, T298S, C311Y, T338P, M342V, M361T, R389Q, N396S, R476Q, R476W
