# Supplementary material for: Genetic and Structure-Function Studies of Missense Mutations in Human Endothelial Lipase
Source: PLoS One. 2013 Mar 25;8(3):e55716. doi: 10.1371/journal.pone.0055716 (PMC3607615; doi:10.1371/journal.pone.0055716)
Supplement: Supporting Information S1 — File S1, The lipid panel in the SLVDS carriers of LIPG T111I missense. File S2, Human EL protein sequence with highlighting all known structural motifs and missense mutations. File S3, Structure-function correlation of all known missense mutations in EL. File S4, Structural close-up of all known missense mutations in EL structural model. File S5, Atomic coordinates for the complete EL homodimer molecular model (separate file, PDB format). File S6, The list of primers used in LIPG mutagenesis. File S7, Western blot of media containing EL. (ZIP) [file pone.0055716.s001.zip › Supporting Files/S-4.pdf]

S – 4. Structural analysis of the missense mutations in EL. A close-up and a 'big picture' are shown for each mutation. A schematic structure of EL is provided here to orient the reader on the structure layout and where the mutations are.

Mutation	Page #
G26S	S5 – 6
E28K	S5 – 7
N52S	S5 – 8
R54C	S5 – 9
P73L	S5 – 10
T111I	S5 – 11
A116T	S5 – 12
G176R	S5 – 13
I239T	S5 – 14
T298S	S5 – 15
C311Y	S5 – 16
T338P	S5 – 17
M342V	S5 – 18
M361T	S5 – 19
R389Q	S5 – 20
N396S	S5 – 21
R476Q	S5 – 22
R476W	S5 – 22

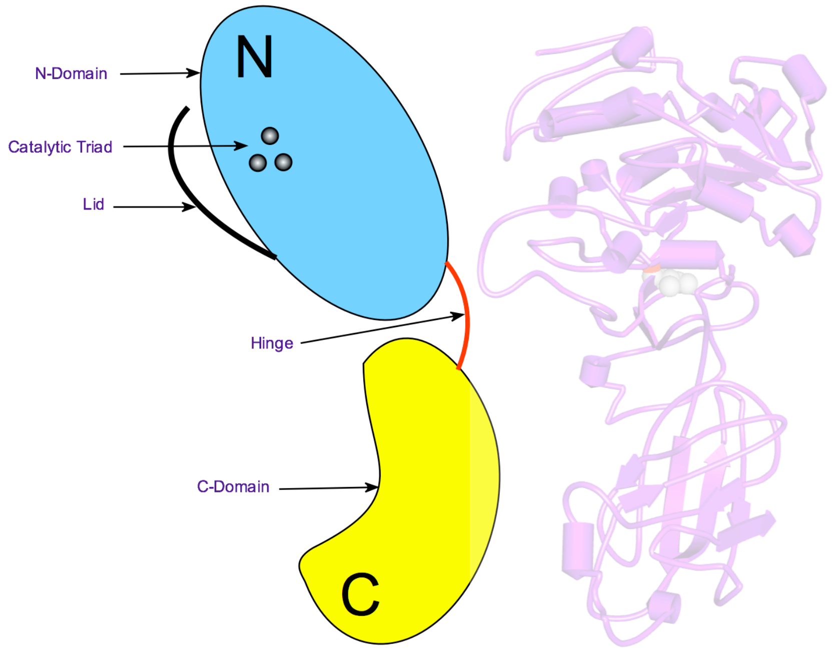

G26S

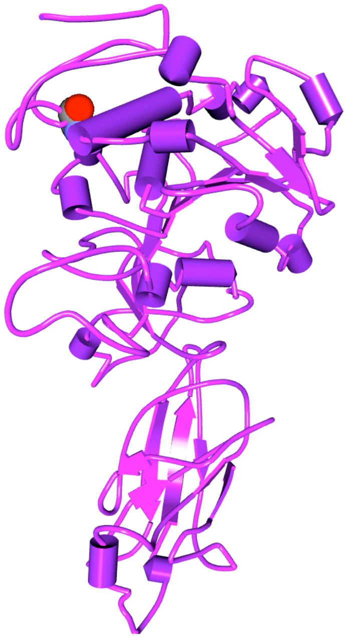

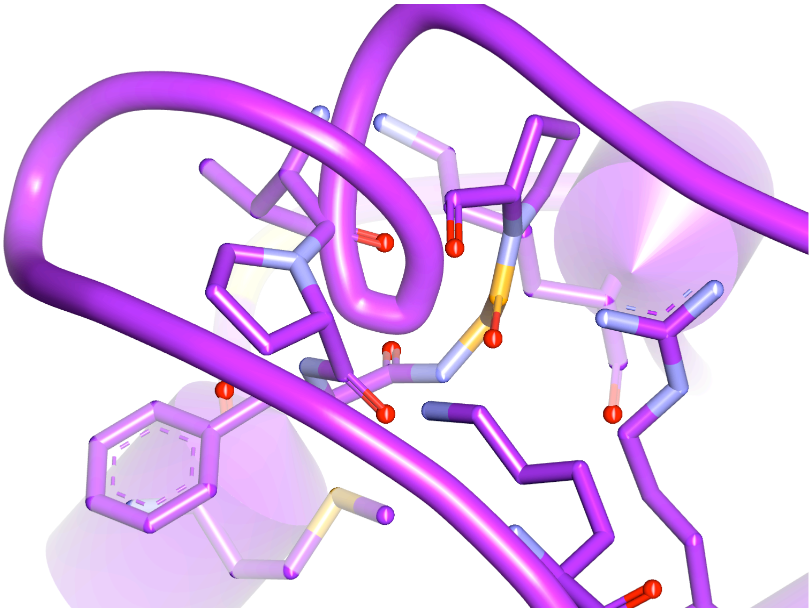

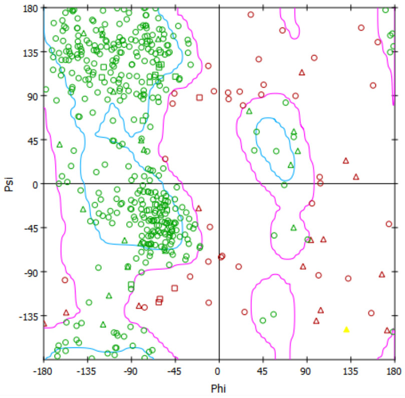

G26S mutation is a substitution of a hydrophobic glycine by a hydrophilic serine, which leads to a decreased degree of variation in allowed ϕ-ψ angles that can result in changes in the N-terminal fold. This mutation resulted in a 39-44% reduction in lipase activity.

E28K

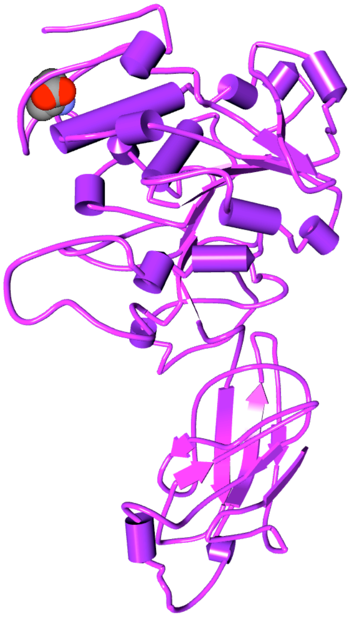

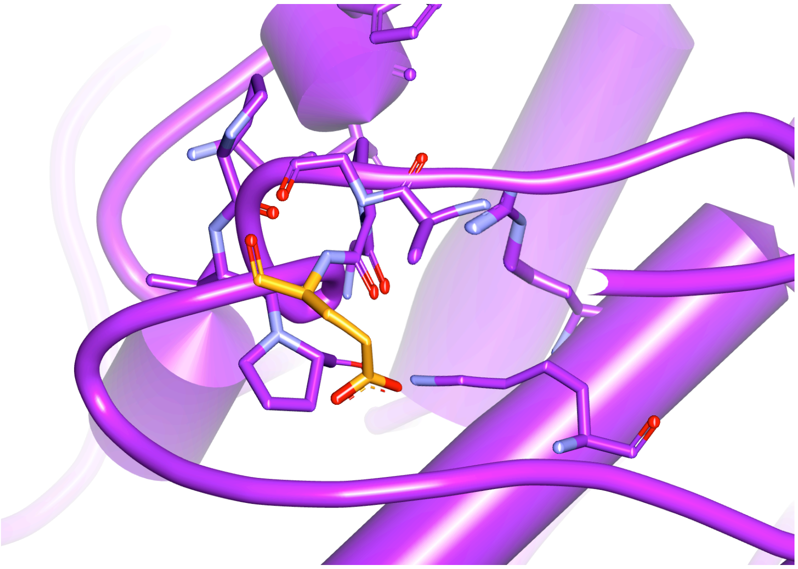

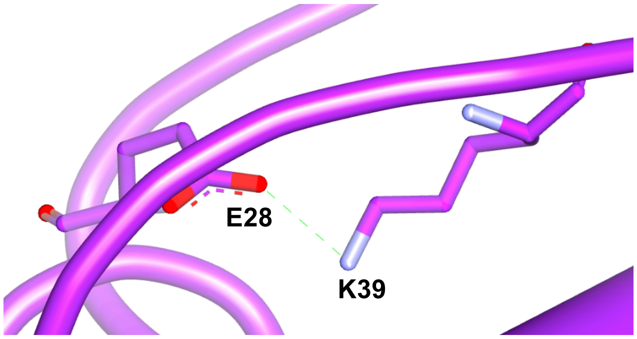

E28K is a substitution of a positive charge, lysine (pK_a=10.0), for a negative charge residue, glutamate (pK_a=4.4), which destroys the salt bridge between E28 and K39 leading to local conformation changes in the N-terminal fold, which may impact the interaction of the mutant with HDL surface. This mutation resulted in a 39-44% reduction in lipase activity.

N52S

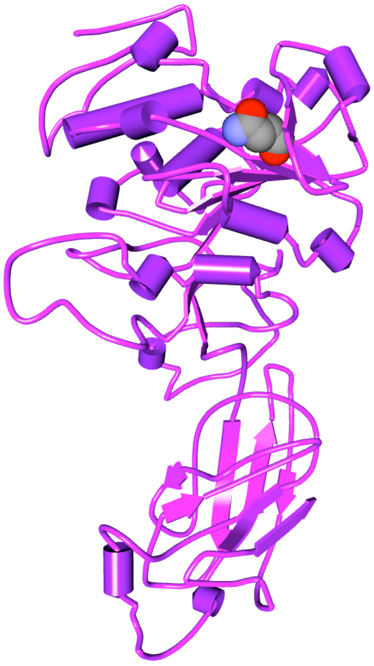

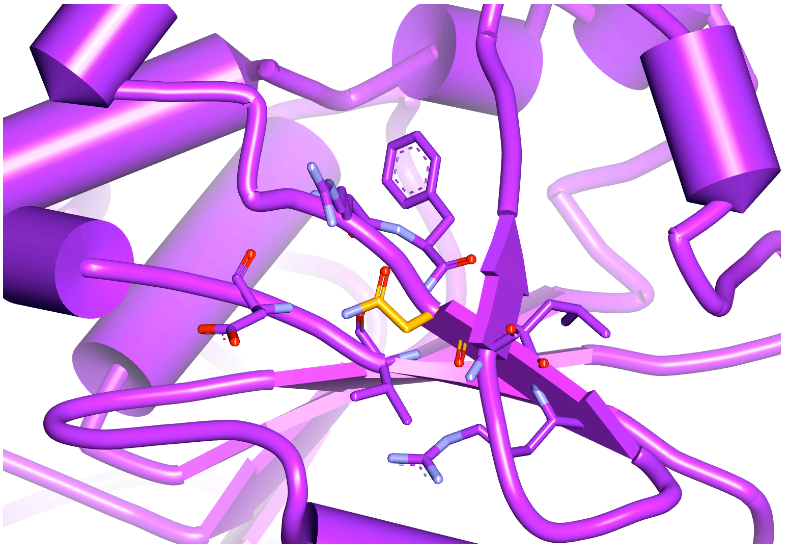

N52S is substitution of serine for asparagine, both of which are hydrophilic. The substitution replaces the amide group in asparagine with the smaller hydroxyl side chain of serine, and results in significant changes in the N-domain conformation. This mutation resulted in a complete loss of lipase activity. However, the exact structural changes as a result of this mutation could not be delineated.

<table>
<tr><th colspan="2">R54C</th></tr>
<tr><td>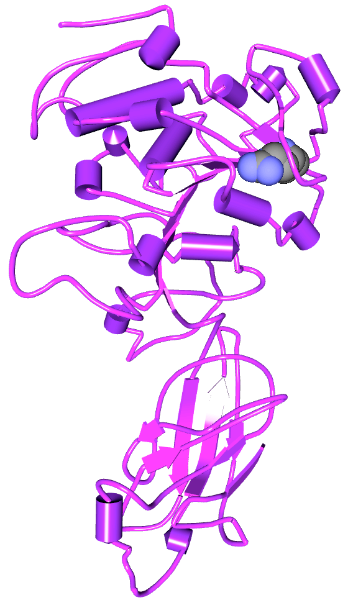</td><td>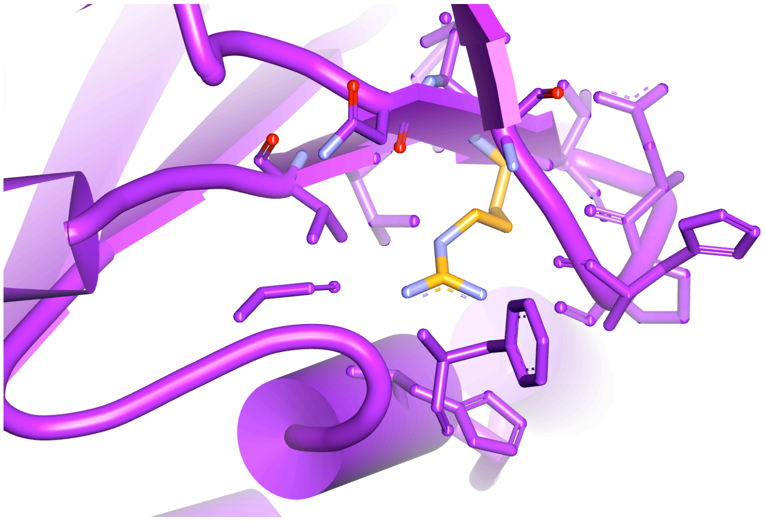</td></tr>
<tr><td colspan="2">R54C is a substitution of a hydrophilic and smaller cysteine for a positively charged arginine, which may contribute to a partial loss of substrate binding specificity (HDL versus other lipoprotein particles). This mutation resulted in an 11-21% reduction in lipase activity.</td></tr>
</table>

P73L

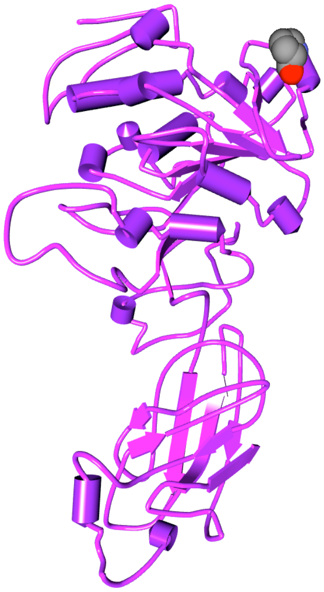

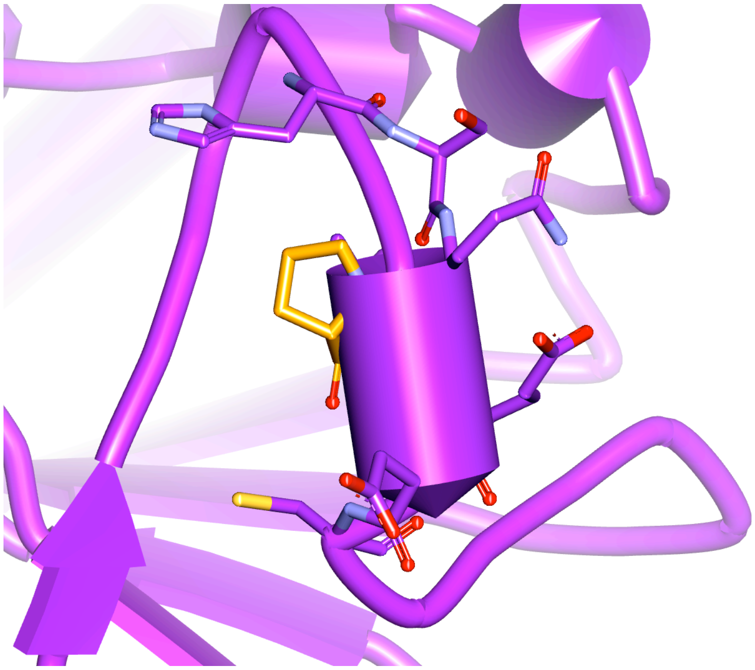

P73L is a substitution of leucine for proline, both of which are nonpolar. Proline is located at the end of a short helix; its occurrence terminating the helix. Substitution of leucine might promote the extension of the helix, thereby changing the fold, which is involved in dimerization of two EL monomers. This mutation resulted in a complete loss of lipase activity.

T111I

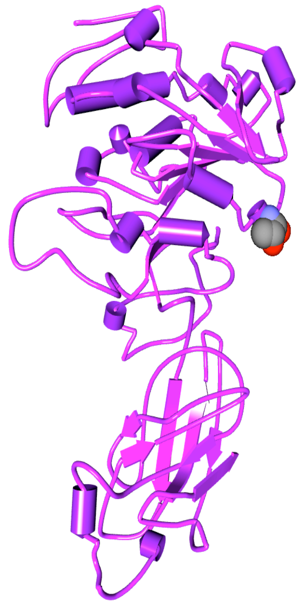

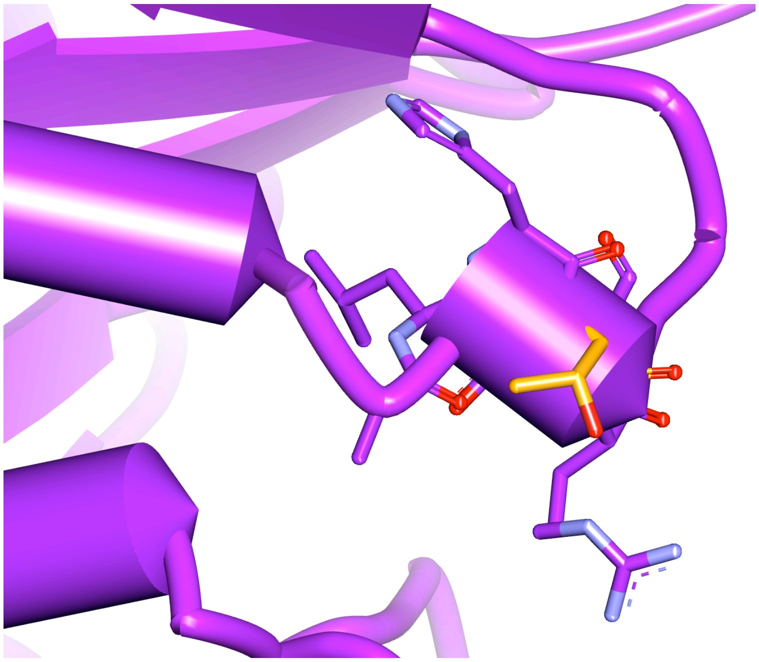

T111I is substitution of isosteres – hydrophobic isoleucine for hydrophilic threonine. This mutation is located at the very end of a helix, which may not be important in substrate interaction or for EL dimerization. However, our genetic association studies suggest this polymorphism is associated with LDL-C and total cholesterol, which may be interpreted as enhanced bridging function in the mutant EL. If this is the case, we cannot correlate the structural impact of this N-domain mutation on the C-domain of EL. This substitution did not impact the lipase activity of the enzyme.

<table>
<tr><th colspan="2">A116T</th></tr>
<tr><td>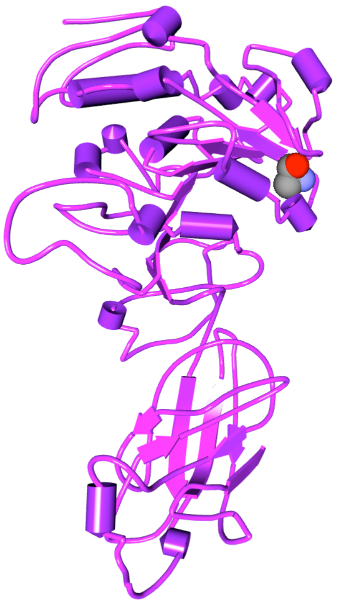</td><td>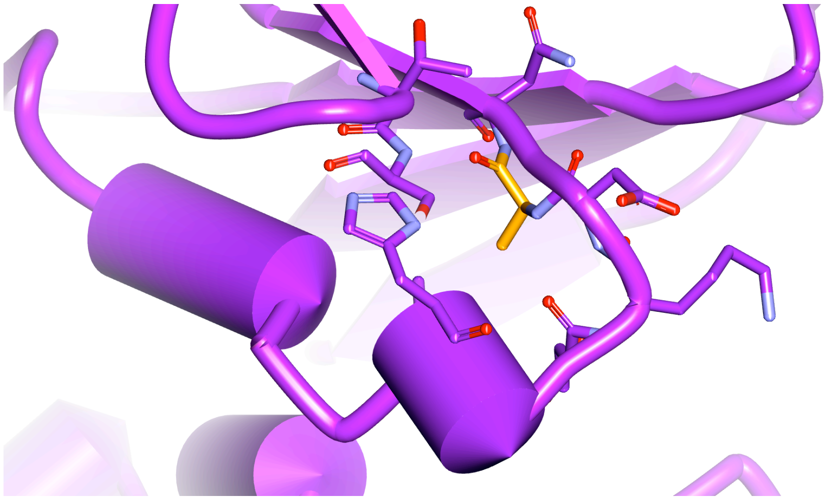</td></tr>
<tr><td colspan="2">A116T is a substitution of polar threonine for hydrophobic alanine. The structure-function slope (Figure 9) predicts about 50% loss of lipase activity for this mutation.</td></tr>
</table>

<table>
<tr><th colspan="2">G176R</th></tr>
<tr><td>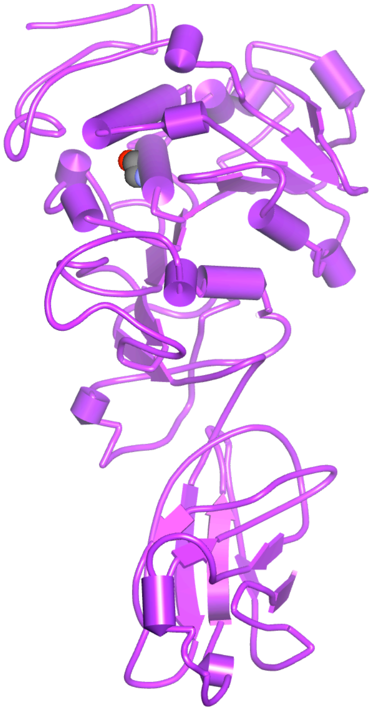</td><td>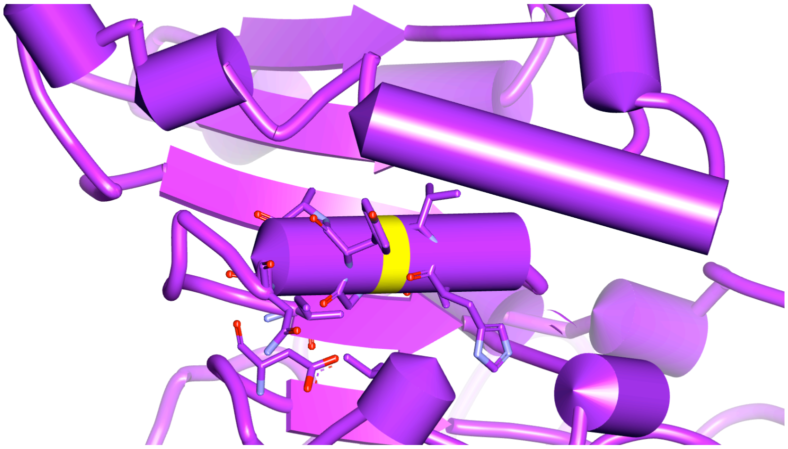</td></tr>
<tr><td colspan="2">G176R is a substitution of positively charged arginine for hydrophobic glycine, which may cause significant conformational changes near the catalytic site. The structure-function slope (Figure 9) predicts about 50% loss of lipase activity for this mutation.</td></tr>
</table>

I239T	
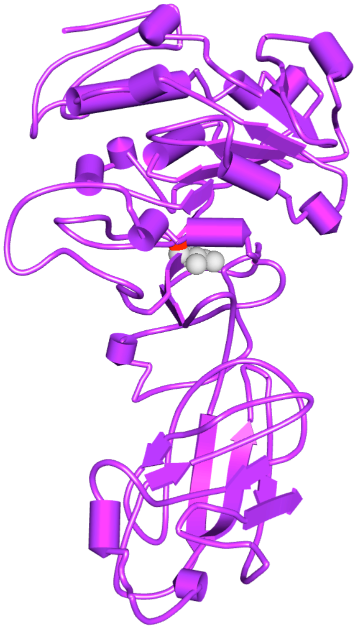	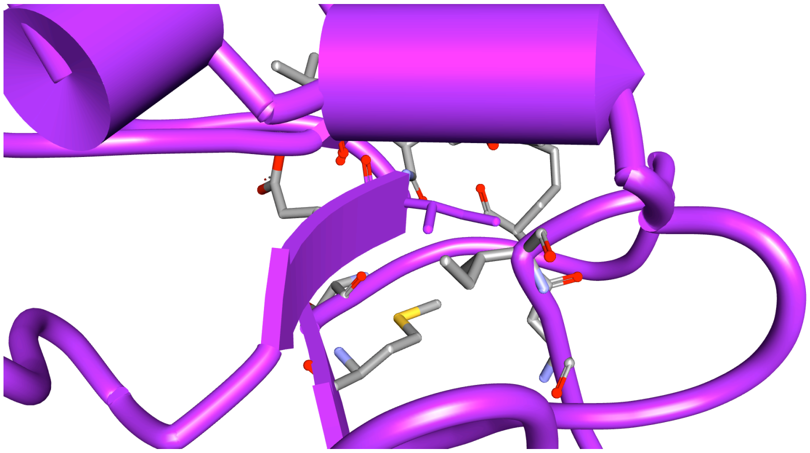
I239T is a substitution of hydrophilic threonine for hydrophobic isoleucine. Similar to G176R, we predict conformational changes near the housing of the catalytic site. The structure-function slope (Figure 9) predicts about 40% loss of lipase activity for this mutation.	

<table>
<tr><td colspan="2">T298S</td></tr>
<tr><td>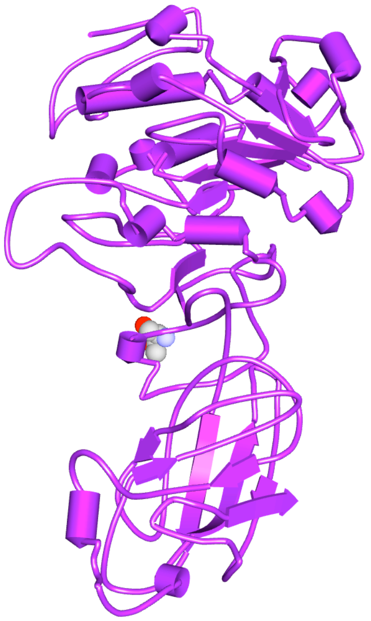</td><td>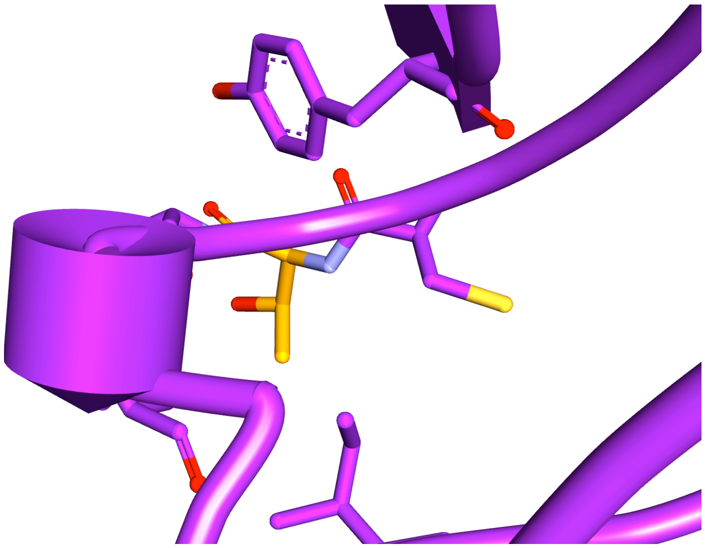</td></tr>
<tr><td colspan="2">T298S is a substitution of hydrophilic serine for hydrophilic threonine. The structure-function slope (Figure 9) predicts about 20% loss of lipase activity, which is compatible with its location and the type of substitution.</td></tr>
</table>

C311Y

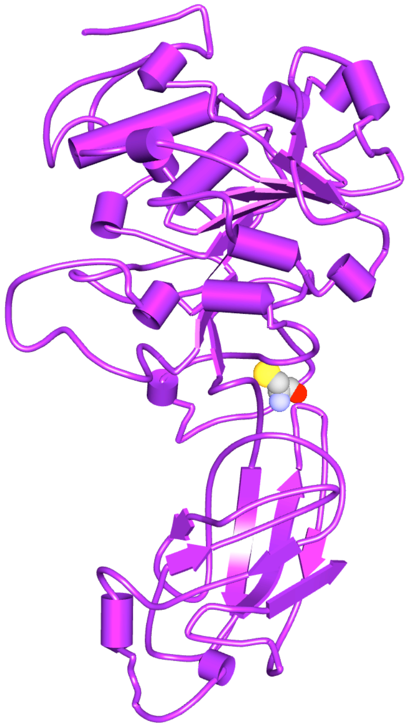

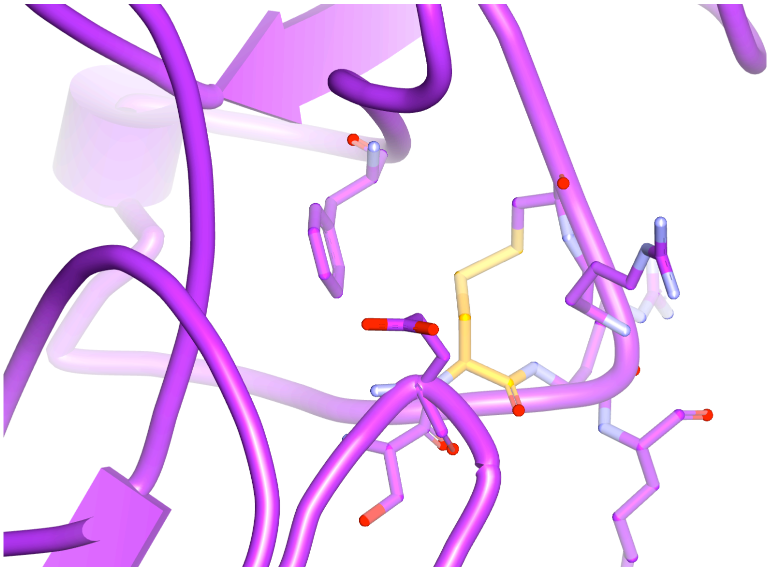

C311Y is substitution of tyrosine for cysteine, both of which are nonpolar residues. However, this substitution disrupts the disulfide bond between cysteine 308 and 311, which would be expected to destabilize the enzyme and impact the heparin-binding motif. We predict this mutation to impact HSPG binding capability of EL, leading to loss of both lipase and bridging functions of EL. However, the structure-function slope (Figure 9) predicts only 10% loss of lipase activity. Functional and population studies will be required to ascertain the impact of this intriguing mutation on EL structure and on HDL-C levels.

T338P

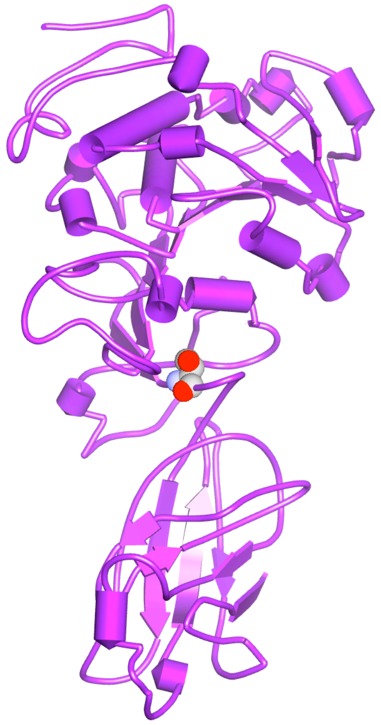

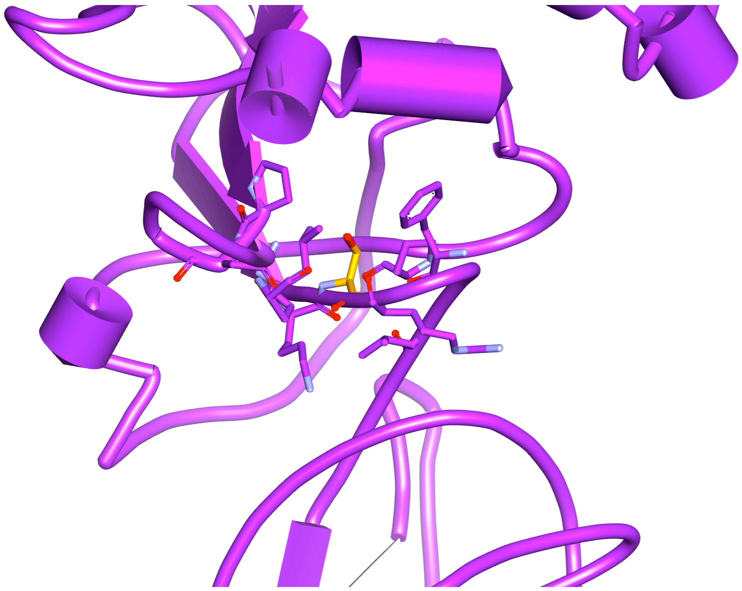

T338P is a substitution of hydrophobic proline for hydrophilic threonine. This residue is located in the hinge loop between the two domains. Furthermore, the threonine dihedral angles (ϕ = -93 and ψ = +176) cannot be adopted by the proline residue, thus this mutation leads to substantial conformational changes in both domains. We predict this mutation impacts both HDL binding and lipase activity. Our lipase assay did not detect any lipase activity for this interesting substitution. It would be interesting to analyze HDL-C levels and the degree of atherosclerosis in subjects with this mutation.

<table>
<tr><td colspan="2">M342V</td></tr>
<tr><td>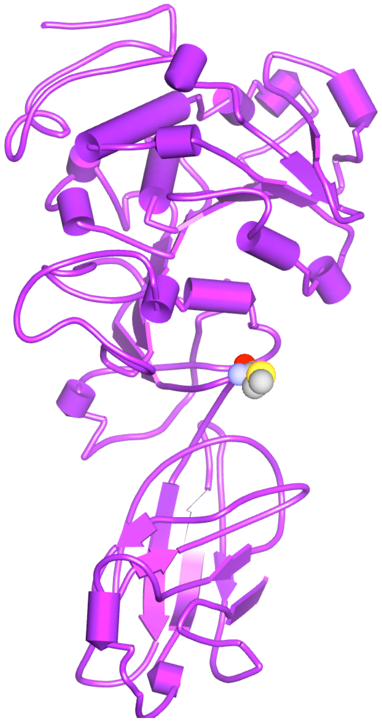</td><td>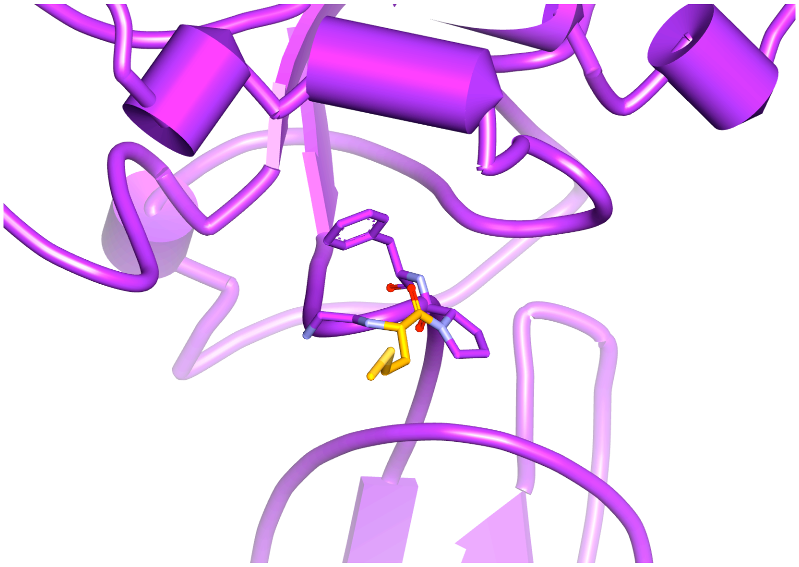</td></tr>
<tr><td colspan="2">M342V is substitution of valine for methionine, both hydrophobic residues, in the hinge loop, which should have a mild impact, if any. The structure-function slope (Figure 9) predicts only 10% loss of lipase activity.</td></tr>
</table>

M361T

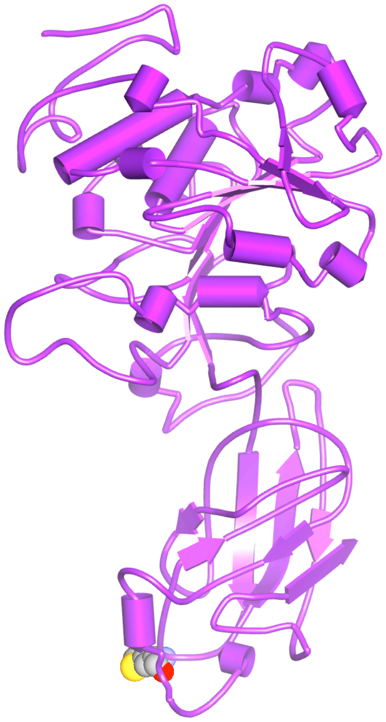

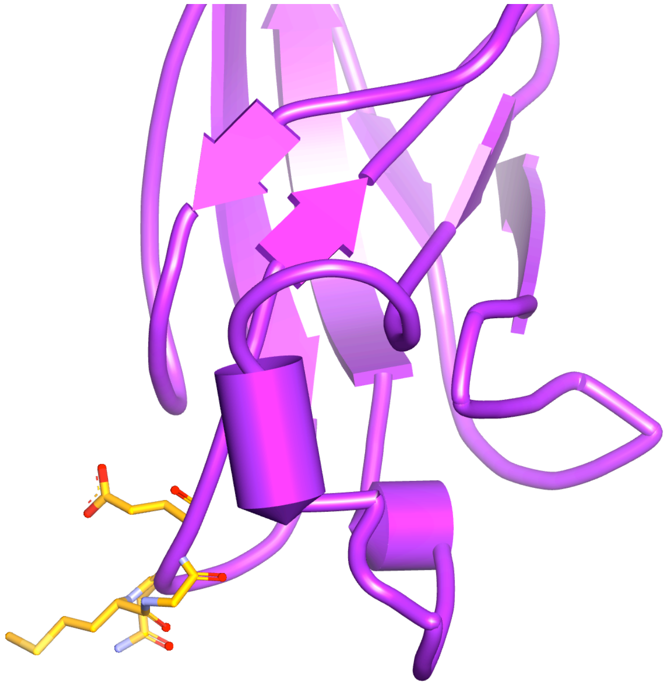

M361T is substitution of hydrophilic threonine for hydrophobic methionine. It is located in a small loop in the PLAT domain. Our lipase assay showed this mutation has no impact on lipase activity of EL.

R389Q

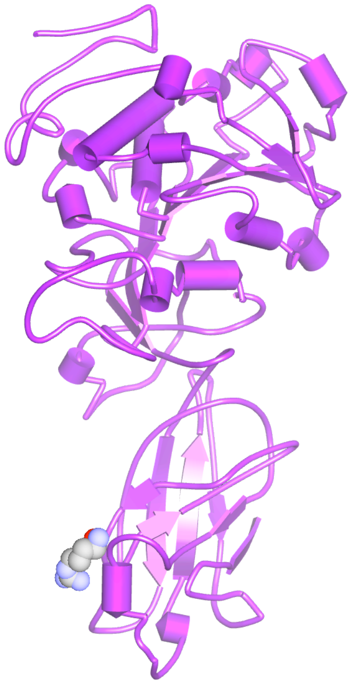

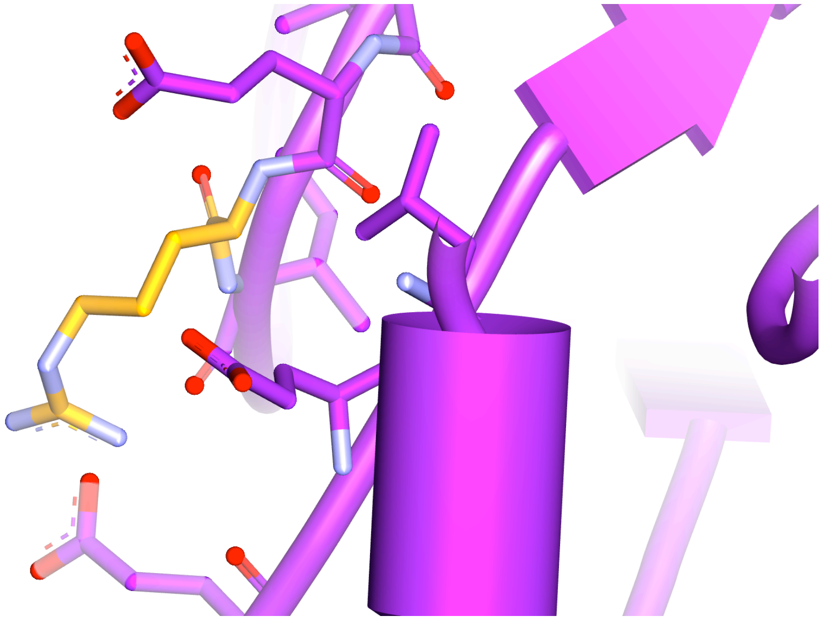

R389Q is substitution of hydrophilic glutamine for the positive charge arginine, which abolishes the salt bridge between R389 and E363. This mutation may impact substrate recognition due to the negative charge not being balanced. Our lipase assay showed 11-21% lipase activity reduction in the mutant. This is yet another mutation for which the effect on function does not seem to match its predicted impact on the structure.

<table>
<tr><td colspan="2">N396S</td></tr>
<tr><td>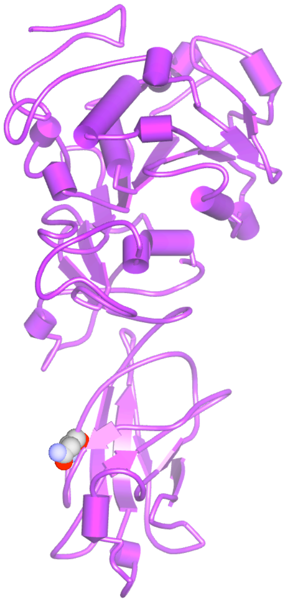</td><td>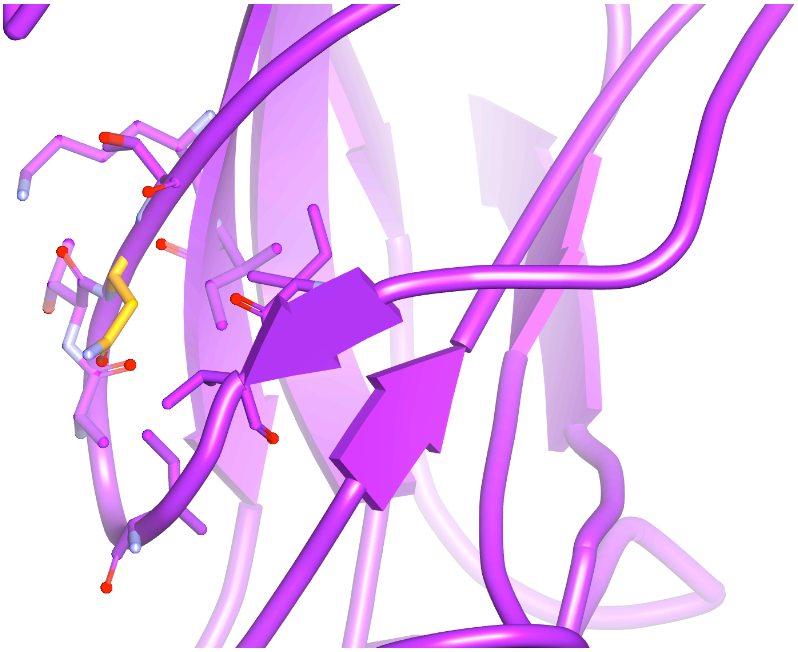</td></tr>
<tr><td colspan="2">N396S is a substitution of serine for asparagine, both hydrophilic. N^{396} is one of five glycosylation sites, and the mutation eliminates this site. Our assays showed this mutation causes 11-21% reduction in lipase activity.</td></tr>
</table>

R476Q , R476W

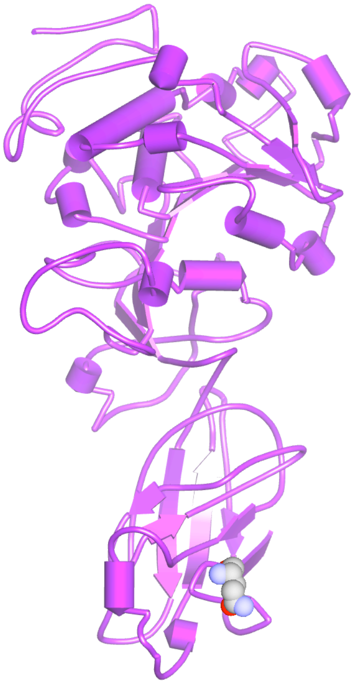

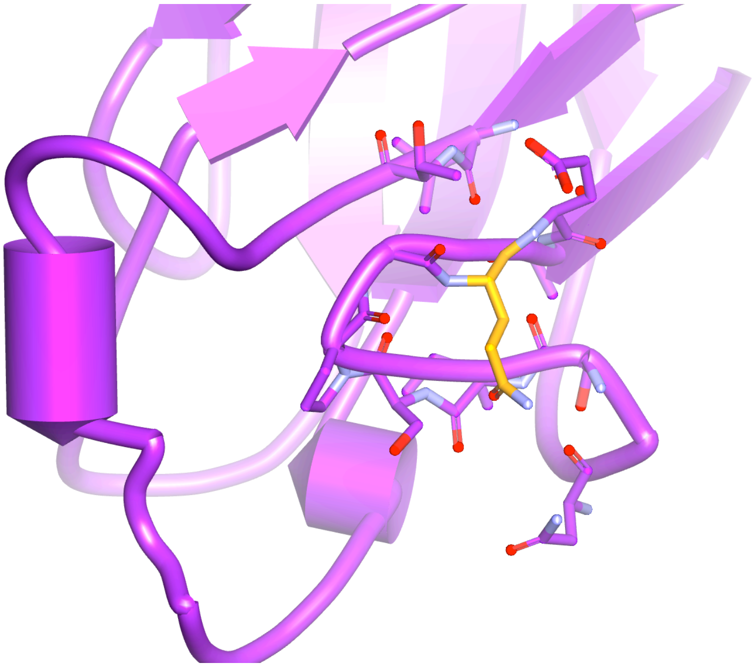

There are two missense mutations known for the positive charge and hydrophobic arginine at position 476. One is R476Q (substitution by hydrophilic glutamine) and the other is R476W (substitution by hydrophobic tryptophan). A substitution of this residue may impact both substrate binding and dimerization. However, the structure-function slope (Figure 9) predicts only an approximately 10% loss of activity for R476W.
