# Supplementary material for: Genetic and Structure-Function Studies of Missense Mutations in Human Endothelial Lipase
Source: PLoS One. 2013 Mar 25;8(3):e55716. doi: 10.1371/journal.pone.0055716 (PMC3607615; doi:10.1371/journal.pone.0055716)
Supplement: Supporting Information S1 — File S1, The lipid panel in the SLVDS carriers of LIPG T111I missense. File S2, Human EL protein sequence with highlighting all known structural motifs and missense mutations. File S3, Structure-function correlation of all known missense mutations in EL. File S4, Structural close-up of all known missense mutations in EL structural model. File S5, Atomic coordinates for the complete EL homodimer molecular model (separate file, PDB format). File S6, The list of primers used in LIPG mutagenesis. File S7, Western blot of media containing EL. (ZIP) [file pone.0055716.s001.zip › Supporting Files/S-6.pdf]

S – 6. The list of the primers used in human *LIPG* mutagenesis and lipase assay experiments

1	*LIPG*-G26S	**F:**	GTACCTTTTAGTCCAGAGGGAC
		**R:**	GTCCCTCTGGACTAAAAGGTAC
2	*LIPG*-E28K	**F:**	GTCCAAAGGGACGGCTGGAAG
		**R:**	CTTCCAGCCGTCCCTTTGGAC
3	*LIPG*-N52S	**F:**	GAGGTTTAGCCTCCGCACCTC
		**R:**	GAGGTGCGGAGGCTAAACCTC
4	*LIPG*-R54C	**F:**	GTTTAACCTCTGCACCTCCAAG
		**R:**	CTTGGAGGTGCAGAGGTTAAAC
5	*LIPG*-P73L	**F:**	CACAGCCAGCTCTTAGAAGAC
		**R:**	GTCTTCTAAGAGCTGGCTGTG
6	*LIPG*-T111I	**F:**	GTCAGCCCTGCACATAAGAGAGA
		**R:**	TCTCTCTTATGTGCAGGGCTGAC
7	*LIPG*-T338P	**F:**	GTACCTAAAACCCCGGGCAG
		**R:**	CTGCCCGGGGTTTTAGGTAC
8	*LIPG*-M361T	**F:**	GTTACAAGAACACGGGAGAAATTG
		**R:**	CAATTTCTCCCGTGTTCTTGTAAC
9	*LIPG*-R389Q	**F:**	ATAGTGGAGCAGATCGAGCAG
		**R:**	CTGCTCGATCTGCTCCACTAT
10	*LIPG*-N396S	**F:**	GAATGCCACCAGCACCTTCCTG
		**R:**	CAGGAAGGTGCTGGTGGCATTC
