# Supplementary material for: Genetic and Structure-Function Studies of Missense Mutations in Human Endothelial Lipase
Source: PLoS One. 2013 Mar 25;8(3):e55716. doi: 10.1371/journal.pone.0055716 (PMC3607615; doi:10.1371/journal.pone.0055716)
Supplement: Supporting Information S1 — File S1, The lipid panel in the SLVDS carriers of LIPG T111I missense. File S2, Human EL protein sequence with highlighting all known structural motifs and missense mutations. File S3, Structure-function correlation of all known missense mutations in EL. File S4, Structural close-up of all known missense mutations in EL structural model. File S5, Atomic coordinates for the complete EL homodimer molecular model (separate file, PDB format). File S6, The list of primers used in LIPG mutagenesis. File S7, Western blot of media containing EL. (ZIP) [file pone.0055716.s001.zip › Supporting Files/S-7.pdf]

S – 7. Comparing the levels of EL protein variants in the HEK-293 media using Coomassie stained SDS-PAGE and immunoblotting techniques with a commercially available antibody. Coomassie blue–stained gel showing similar overall protein levels present in the EL expressing HEK-293 media (A). Lane 1 is a molecular weight ladder, lanes 2 and 3 are media from HEK-293 cells producing wild type EL and the EL mutant [T338P], respectively. Western blot of EL in HEK-293 media showing protein bands, though in a 'smiley' form, at extreme overexposure times (B). Wild type EL and the mutant EL [T338P] were blotted from a gel similar to panel A using an anti-EL polyclonal antibody and exposed for 1 to 30 minutes. Mature EL protein (480 aa) with glycosylation has a molecular weight of 68 kDa. Both Coomassie-stained and Western blot gels show a band seems corresponding to 75 kDa marker vicinity.

In our opinion, these results are not convincingly conclusive in observing and quantifying the EL protein variants in the media. It is not surprising that a highly active enzyme such as EL could still show solid activity at levels that would be poorly detectable using these approaches. Thus, we conclude the levels of the EL wild type and mutant in the media were just too low to visualize and quantify reliably. That said, the expression protocol for the WT EL and the MT EL was identical; and therefore, we do not expect much variation from experiment to experiment and so we feel confident that we can interpret the decrease in activity observed in lipase assays and MS/GC experiments as an effect of the mutation on either the activity or stability of the protein.

**A. Coomassie-stained Gel**	**B. Western blot Gel**
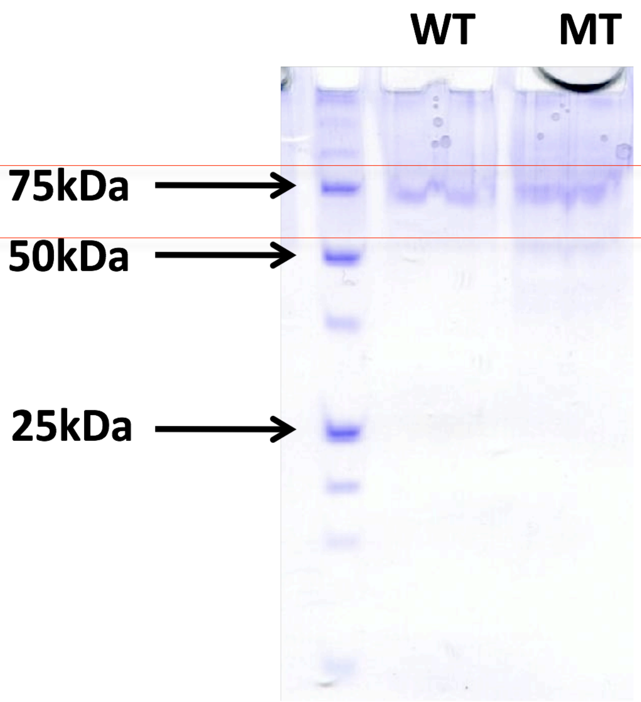	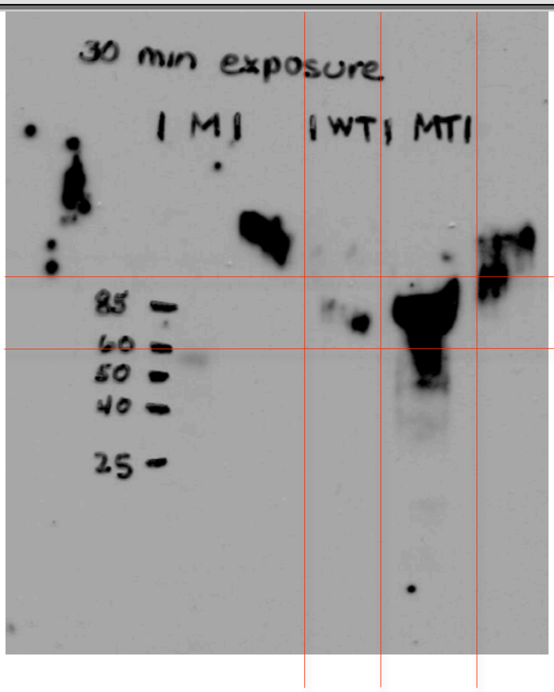
